# Comprehensive Mapping of Functional Enhancers in Chinese Hamster Ovary Cells

**DOI:** 10.1002/bit.70076

**Published:** 2025-10-03

**Authors:** María Santos, Yusuf B. Johari, Laura Biggins, Natalie C. Elliott, Stefan Schoenfelder, Mounika Boddireddy, Daniel K. Fabian, Michael Anbar, Peter M. O'Callaghan, Peter J. Rugg‐Gunn

**Affiliations:** ^1^ Epigenetics Programme Babraham Institute Cambridge UK; ^2^ Lonza Biologics R&D Cambridge UK; ^3^ Bioinformatics Group, Babraham Institute Cambridge UK; ^4^ Loke Centre for Trophoblast Research University of Cambridge Cambridge UK; ^5^ Cambridge Stem Cell Institute University of Cambridge Cambridge UK

**Keywords:** CHO cells, cis‐regulatory element, enhancer, gene regulation, STARR‐seq, transcription factor

## Abstract

Chinese hamster ovary (CHO) cells are the leading mammalian system for recombinant therapeutic protein production. However, optimizing transgene expression remains challenging due to the limited understanding of the regulatory mechanisms controlling gene expression in CHO cells. Towards overcoming this barrier, here we provide a systematic characterization of cis‐regulatory elements in CHO cells. Using genome‐wide STARR‐seq, a high‐throughput method for quantifying enhancer strength, we identified regions with enhancer activity in the CHO cell genome. By integrating these data with ATAC‐seq and histone modification profiles, we were able to characterize the chromatin state of these regions. Our analysis revealed thousands of newly identified enhancer sequences. The most active sequences could drive transgene expression at levels similar to or higher than strong viral enhancers. Notably, half of the regions found to have enhancer activity were within inaccessible chromatin in their native context. We observed that accessible enhancers were primarily near to transcriptional start sites and associated with ubiquitously‐expressed genes, whereas inaccessible enhancers were predominantly intergenic and associated with tissue‐specific genes. Additionally, through a deep‐learning‐based approach ETS and YY1 transcription factor (TF) binding motifs were identified as key determinants of enhancer identity and strength. Disrupting YY1 binding motifs led to reduced enhancer activity, thereby highlighting the importance of YY1 as a transcriptional activator in CHO cells. Our study demonstrates the first comprehensive map of functionally‐validated enhancers in CHO cells and generates new insights into gene regulation and the role of TFs in determining enhancer strength. This study helps to lay the foundation for strategic engineering of CHO cell transcriptional networks to achieve enhanced biopharmaceutical production.

## Graphical abstract

In this study, the authors identified regions with enhancer activity in the CHO cell genome using genome‐wide and targeted STARR‐seq. The analysis revealed thousands of newly identified enhancer sequences, where some of the most active sequences could drive transgene expression at levels similar to or higher than strong viral enhancers. The study demonstrates a comprehensive map of functionally‐validated enhancers in CHO cells and generates new insights into gene regulation and the role of transcription factors in determining enhancer strength.

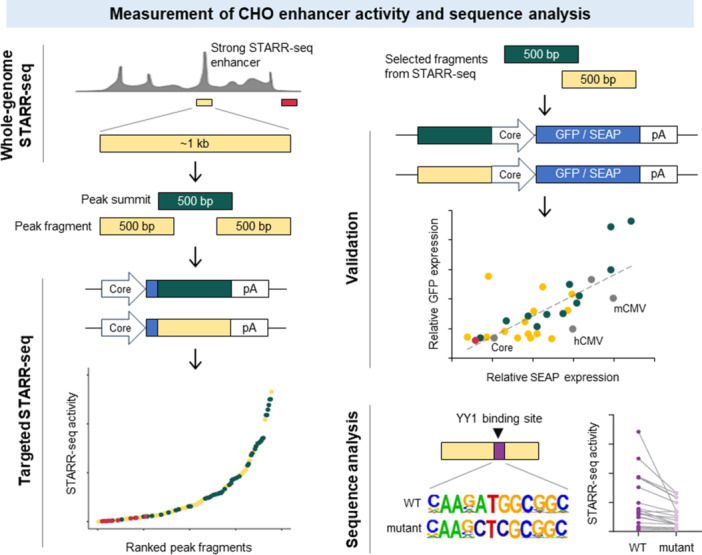

**Abbreviations**
CHO: Chinese hamster ovary
CMV: cytomegalovirus
GFP: green fluorescent protein
GO: gene ontology
GS: glutamine synthetase
hCMV: human cytomegalovirus
mAb: monoclonal antibody
mCMV: murine cytomegalovirus
RPKM: reads per kilobase per million mapped reads
SEAP: secreted alkaline phosphatase
TF: transcription factor
TPM: transcripts per million
TSS: transcription start site
TTS: transcription termination site
UPR: unfolded protein response
UTR: untranslated region

## 1 Introduction

Chinese hamster ovary (CHO) cells are the predominant host system to produce therapeutic recombinant proteins. Approximately 90% of mammalian cell‐derived biopharmaceuticals approved between 2008 and 2022, along with the majority of commercially available monoclonal antibodies (mAbs), are produced using CHO cells (Walsh and Walsh 2022). Although CHO expression platforms (e.g., the glutamine synthetase (GS) selection system), have been extensively optimized across the whole upstream process from DNA design to fed‐batch culture, the growing patient‐led demand for ever more complex multi‐chain biotherapeutic proteins means that expression systems must continue to evolve and improve. As a critical “substrate” in the manufacturing of such proteins, the DNA expression vector plays an important part in influencing the overall cost of goods, based on the interplay between the product harvest titer at commercial scale and the protein quality, among other factors. This makes the expression vector an important target for optimization (O'Callaghan and Johari 2025).

Optimization of the regulatory sequences driving vector transgene expression is a promising strategy for increasing the yield of recombinant therapeutic proteins in CHO cell lines. Typical CHO expression vectors are largely based on regulatory sequences derived from viruses, and although sequences such as the cytomegalovirus (CMV) promoter/enhancer have been hugely beneficial in the industry by driving up high titers across multiple products for many years (Boshart et al. 1985; Rotondaro et al. 1996; Bebbington 1991), opportunities for further improvements remain. In particular, regulatory sequences that combine optimal transcriptional output for a wide range of therapeutic proteins encoded by multiple (≥ 3) product genes within a single plasmid, together with long‐term expression stability and reduced risk of cellular stress (i.e., no/minimal unfolded protein response [UPR]), would be of significant interest to the industry. As our knowledge of both the linear genomic sequence and also the genetic regulatory network and epigenome of the CHO host cell line has improved (Bevan et al. 2021; Hilliard and Lee 2021; Lewis et al. 2013), bioprocessing scientists can now reasonably ask if the CHO genome itself can become a source of new regulatory sequences to improve expression vector design. Candidate sequences to improve the transcriptional output from conventional expression vectors could include strong enhancer elements isolated from the host cell genome.

Enhancers are cis‐regulatory elements that induce gene expression of their target gene(s) by activating transcription initiation at promoters (Panigrahi and O'Malley 2021). These regulatory sequences consist of dense clusters of binding sites for transcription factors (TFs) that facilitate the assembly of the transcriptional machinery at their target promoters. While sequence‐encoded instructions determine intrinsic potential of a specific sequence to act as an enhancer, other layers of regulation, such as chromatin accessibility or TF availability, further modulate enhancer activity and shape the gene expression pattern in a specific cell type. If CHO‐derived regulatory sequences were repurposed in novel expression vector designs, what benefits could we expect to achieve? We may hypothesize that in comparison to highly active and constitutive promoter/enhancers such as CMV, strong endogenous CHO enhancers may preferentially interact with the wider CHO transcriptional network at points of stable vector integration into the genome. They may also allow better coordination of recombinant gene expression in line with wider “state” changes to the host cell, for example as cells simultaneously cope with both the increased metabolic burden after vector transfection plus stringent selection pressure (e.g., removal of glutamine from the media), or as cells transition across a fed‐batch culture process.

Despite the promising strategy of using CHO‐derived enhancers to optimize expression vector designs, the precise detection of these enhancers remains highly challenging (Smith et al. 2023). Typically, genomic enhancers are identified based on chromatin features, such as accessible chromatin regions (Thurman et al. 2012), acetylation of lysine 27 of histone H3 (H3K27ac) and methylation of lysine 4 (H3K4me1) (Heintzman et al. 2007). Techniques like ATAC‐seq and ChIP‐seq can screen the genome for these features and predict the location of putative enhancers, but these assays cannot directly measure enhancer activity or strength. Thus, endogenous enhancers may be missed or erroneously identified in studies that rely on these methods alone. As an alternative, STARR‐seq is an incisive method that allows genome‐wide quantification of enhancer activities by inserting genomic fragments downstream of a minimal promoter within a reporter plasmid (Arnold et al. 2013). The enhancer capacity of each fragment is then determined by measuring the relative abundance of its corresponding transcript in the cell. STARR‐seq has been used to identify and quantify enhancer activity in different human and mouse cell lines (Barakat et al. 2018; Muerdter et al. 2018; Vanhille et al. 2015) as well as in vivo tissues (Chan et al. 2023).

In CHO cells, studies on regulatory elements have been mostly limited to individual loci (Chen et al. 2013) or have systematically explored their presence in the genome using histone modifications, chromatin accessibility or spatial chromatin configuration (Bevan et al. 2021; Feichtinger et al. 2016; Lee et al. 2021). Thus, our *direct* knowledge of the genome‐wide CHO enhancer landscape and the functional impact of enhancer‐mediated gene regulation in CHO cells is incomplete. Furthermore, not all of the putative enhancers identified to date have been fully tested and functionally validated, for example within recombinant protein expression vectors. Where the CHO genome has been a source of novel promoters and/or enhancers identified to potentially boost recombinant protein expression, the overall throughput of candidate sequence testing has been extremely low, limiting the chances of finding truly novel sequences with unique properties (Nguyen et al. 2019). These data and knowledge gaps mean that a repository of validated CHO enhancer sequences is not currently available for high‐throughput screening as ‘building blocks’ in improved expression vector designs, and also means that we do not know how well any predicted enhancer sequences would interact with non‐cognate promoters (Lorberbaum and Barolo 2015)—thereby holding back the development of more rational strategies for fine‐tuning gene expression patterns using endogenous CHO sequences.

In this study, our aim was to better understand the regulatory properties controlling gene expression in CHO cells through defining and characterizing regions with enhancer activity in the CHO cell genome. Using STARR‐seq, we systematically identified and quantified the activity of enhancers genome‐wide. We then investigated whether a subset of these enhancers could be repurposed to induce recombinant protein expression, and further characterized them in their endogenous genomic context. Our results indicate that the CHO host cell line itself may be a source of regulatory DNA sequences that could potentially be used for recombinant protein expression. Furthermore, our findings additionally pinpointed specific TFs, particularly those of the ETS family and YY1, as key mediators of enhancer activity in CHO cells.

## 2 Materials and Methods

### 2.1 CHO Cell Culture

The GS Xceed® CHOK1SV GS‐KO® cell line (Lonza) was cultured in suspension using CD‐CHO medium supplemented with 6 mM l‐glutamine (Thermo Fisher Scientific). Cells were maintained at 36.5°C, 140 rpm, under 5% CO_2_ and 85% humidity. Cells were sub‐cultured every 3‒4 days by seeding at 2 × 10^5^ cells/mL. Cell viability and viable cell density were measured using a Vi‐CELL Analyzer (Beckman Colter).

### 2.2 IFN‐Related Genes Expression Quantification

20 × 10^6^ CHOK1SV GS‐KO® cells (at 1 × 10^6^ cells/mL) were transiently transfected in Erlenmeyer flasks with 40 µg of pmaxGFP vector (Lonza) and 100 µL of PEI MAX Transporter 5 Transfection Reagent (1 mg/mL; Polysciences), or only 100 µL of PEI (mock transfection). At 24 h and 48 h posttransfection, cells were lysed, and total RNA was extracted using RNeasy miniprep kit (Qiagen). 2 µg total RNA was reverse‐transcribed using Superscript II (Thermo Fisher Scientific) and Oligo(dT)_18_ primer (Thermo Fisher Scientific) according to manufacturer's instructions. cDNA was diluted (1:5) and 2 µL was used for qPCR with 6 µL JumpStart Taq ReadyMix (Sigma‐Aldrich) and 0.4 µL gene specific forward and reverse qPCR primers (10 µM; Supporting Information Table S1) in a total reaction volume of 10 µL. Ct values for each target gene were normalized to *Akr1a1* using the 2^–∆∆Ct^ method. The fold change of ∆∆Ct values for transfections with and without DNA was calculated and displayed in log_2_. Experiment was performed in three biologically independent replicates.

### 2.3 GFP and SEAP Reporter Assays

pmaxGFP vector was used as a backbone. A STARR‐seq enhancer or an inactive sequence, followed by a murine CMV (mCMV) core promoter (‒101 to + 1 relative to the transcriptional start site [TSS]) was cloned upstream of the green fluorescent protein *(GFP)* gene (Supporting Information Figure S1). mCMV (492 bp) and human CMV (hCMV; 598 bp) promoters cloned upstream of the *GFP* gene were used as references of strong expression induction. The mCMV core promoter was used to assess basal expression level, while a promoterless vector served as control for background subtraction. 10 × 10^6^ CHOK1SV GS‐KO® cells (at 1 × 10^6^ cells/mL) were transiently transfected in TubeSpin Bioreactor tubes (TPP) with 20 µg of vector and 50 µL of PEI. Transfected cells were cultured for 48 h at 36.5°C, 230 rpm, under 5% CO_2_ and 85% humidity. GFP expression was quantified 48 h posttransfection using a Cytek Guava easyCyte flow cytometer. GFP expression levels are shown as the percentage of median GFP expression relative to the full‐length mCMV maxGFP vector. To create the secreted alkaline phosphatase (SEAP) reporter vector, the *GFP* gene was removed and replaced with a chemically synthesized *SEAP* gene (Thermo Fisher Scientific). SEAP concentration was measured 48 h posttransfection using the SensoLyte pNPP Alkaline Phosphatase Assay kit (AnaSpec) according to the manufacturer's instructions. SEAP expression levels are shown as the percentage of SEAP titer relative to the full‐length mCMV SEAP vector. All experiments were performed in two biologically independent replicates.

### 2.4 Optimization of STARR‐seq Cell Harvesting Time

To optimize the cell harvesting time after transfection of the STARR‐seq plasmid library, we ran a pilot experiment comparing STARR‐seq activity quantification in cells harvested at 6 h versus 24 h posttransfection. For this experiment, we included 76 synthetic sequences (eBlocks Gene Fragments, IDT; Supporting Information Table S2). These sequences comprised 56 putative enhancers based on chromatin‐state, 10 strong enhancers, and 3 inactive sequences previously identified in mouse cell lines (Vanhille et al. 2015). Additionally, we included 7 negative controls, consisting of 4 random nucleotide sequences and 3 sequences from gene deserts in the CHO genome that overlapped with ATAC‐seq negative regions. STARR‐seq activity for these fragments was measured, with cells harvested at 6 h and 24 h posttransfection. To assess signal‐to‐noise ratio, we defined a set of high‐confidence positive regions (*n* = 14) based on GFP assays or previously reported strong enhancers in mouse cell lines. Signal‐to‐noise ratio for each positive region was calculated as the fold‐change of STARR‐seq activity over the mean STARR‐seq activity of the negative control regions.

### 2.5 STARR‐seq Enhancer Length Optimization

For optimization of whole‐genome STARR‐seq peak length, we selected the 50 most active peaks identified in the whole‐genome STARR‐seq experiment. Each selected peak was *in silico* fragmented in 500 bp bins centered on the peak summit and with 200 bp overlap among flanking bins. As negative controls we used 500 bp sequences from gene deserts or random nucleotide sequences. In total, 186 gene fragments were designed, of which 159 were successfully synthesized (Twist Bioscience). STARR‐seq activity for these fragments was measured.

### 2.6 Whole‐Genome and Targeted STARR‐seq

STARR‐seq was performed following published protocols (Muerdter et al. 2018; Neumayr et al. 2019) with some modifications.

#### 2.6.1 Insert DNA

For targeted STARR‐seq, DNA pools with equimolar representation of synthetic gene fragments including flanking adapters (insert_5’_adapter: 5’‐ACACTCTTTCCCTACACGACGCTCTTCCGATCT‐3’, insert_3’_adapter: 5’‐AGATCGGAAGAGCACACGTCTGAACTCCAGTCAC‐3’) and STARR‐seq vector homology arms (insert_5’_homology_arm: 5’‐TAGAGCATGCACCGG‐3’; insert_3’_homology_arm: 5’‐TCGACGAATTCGGCC‐3’) were generated. For whole‐genome STARR‐seq, the library inserts (700‒900 bp) were generated as previously described (Neumayr et al. 2019). Briefly, genomic DNA was extracted from CHOK1SV GS‐KO® cultured cells using Monarch Genomic DNA Purification kit (NEB). Genomic DNA (50 µg) was sheared by sonication (E220 Focused‐ultrasonicator, Covaris) to achieve an average fragment size of 800 bp. Fragmented DNA was then run in an 1% agarose gel, and fragments of 800 bp ( ± 100 bp) were purified using QIAquick Gel Extraction kit (Qiagen). Size‐selected DNA was then end‐repaired and ligated with the NEBNext hairpin adapter (NEBNext Ultra II DNA Library Prep Kit for Illumina, NEB; NEBNext Multiplex Oligos for Illumina, NEB). The adapter‐ligated DNA was purified twice with 1.8X and 0.8X AMPure XP beads (Beckman Colter) and eluted in 100 µL and 20 µL of 10 mM Tris‐Cl, pH 8.5, respectively. Purified adapter‐ligated DNA library inserts were PCR amplified with the STARR‐seq library cloning forward (5’‐TAGAGCATGCACCGGACACTCTTTCCCTACACGACGCTCTTCCGATCT‐3’) and reverse (5’‐GGCCGAATTCGTCGAGTGACTGGAGTTCAGACGTGTGCTCTTCCGATCT‐3’) primers (Arnold et al. 2013), which added 15 bp vector homology sequence to both ends. In total, 30 PCR reactions were performed (program: 98°C for 45 s, 5 cycles of [98°C for 15 s, 65°C for 30 s, 72°C for 45 s], 72°C for 120 s), using 1 µL of adapter‐ligated DNA per reaction. Reactions were pooled (10 reactions per clean‐up), cleaned up with 0.8X AMPure XP beads (Beckman Colter) and eluted in 100 µL 10 mM Tris‐Cl, pH 8.5. A second purification was done using 3 reactions and a QIAquick PCR purification kit (Qiagen), eluting in 50 µL 10 mM Tris‐Cl, pH 8.5 per reaction. Eluates were pooled together.

#### 2.6.2 Screening Vector

A novel core mCMV STARR‐seq screening vector was designed and synthesized. This screening vector included the core mCMV promoter upstream of an intron, a truncated form of maxGFP, and the STARR‐seq fragments cloning site (Supporting Information Figure S2). To address the possibility that STARR‐seq transcripts might originate from the Ori sequence or the core promoter (Muerdter et al. 2018), we synthesized a STARR‐seq screening vector with Ori serving as the core promoter. This control vector was used as a reference for accurate quantification of transcript levels in the target STARR‐seq approach.

#### 2.6.3 STARR‐seq Plasmid Library Cloning

The screening vector was linearized by digestion with AgeI and SalI enzymes (NEB) for 2 h, followed by agarose gel electrophoresis, QIAquick gel extraction (Qiagen), and clean‐up with QIAquick PCR purification (Qiagen) and MinElute PCR purification (Qiagen). Synthetic or genomic fragments were cloned into the linearized vector at 2:1 molar ratio using In‐Fusion HD Cloning (Takara) in a total reaction volume of 30 µL and 400 µL (10 µL per reaction) for the targeted and whole‐genome approach, respectively. The cloning reactions were pooled, purified with MinElute PCR purification kit (Qiagen) and eluted in 12.5 µL of 10 mM Tris‐Cl, pH 8.5. Eluates were pooled together. MegaX DH10B Electrocomp cells (Thermo Fisher Scientific) were transformed with 2.5 µL of DNA per aliquot (20 µL) according to the manufacturer's protocol. For targeted screens, 3 aliquots of cells were used, and for genome‐wide STARR‐seq, 20 aliquots of bacteria were used. After 1 h of recovery at 37°C, transformation reactions were pooled. For targeted approaches, 1.5 mL of the bacterial culture was transferred to 2 L of LB broth with ampicillin (LB/Amp; 100 µg/mL) and grown overnight. For the whole‐genome approach, 18 mL of bacterial culture was seeded in 12 L of LB/Amp. Aliquots of the transformation were plated on LB/Amp plates to estimate the transformation efficiency. Plasmid library was purified using either Plasmid Plus Maxi kit (Qiagen) or Plasmid Plus Giga kit (Qiagen). To sequence the untransfected STARR‐seq plasmid library, Illumina‐compatible sequencing libraries were generated in a PCR (program: 98°C for 45 s, 5 cycles of [98°C for 15 s, 65°C for 30 s, 72°C for 45 s], 72°C for 120 s) with the KAPA HiFi Hot Start Ready Mix and NEBNext Multiplex Oligos for Illumina (NEBNext Multiplex Oligos for Illumina, NEB).

#### 2.6.4 STARR‐seq Screening

For the targeted screens, 20 × 10^6^ CHOK1SV GS‐KO® cells were transfected per screen. For genome‐wide STARR‐seq, 200 × 10^6^ CHOK1SV GS‐KO® cells were transfected per screen. Cells were transfected with 2 µg of STARR‐seq plasmid library and 5 µL of PEI (1 mg/mL) per million cells. Genome‐wide STARR‐seq screens were performed in duplicate. 6 h (or 24 h for the harvesting time optimization experiment) after transfection, cells were lysed, and total RNA was extracted using RNeasy Mini or RNeasy Maxi (Qiagen) kits. PolyA RNA was isolated using 2X Dynabeads Oligo(dT)_25_ beads (Thermo Fisher Scientific) treated with TurboDNase (Thermo Fisher Scientific) and purified with RNeasy MinElute Cleanup kit (Qiagen). Reverse transcription was performed using SuperScript III Reverse Transcriptase (Thermo Fisher Scientific) and a gene‐specific primer (Supporting Information Table S1). cDNA was treated with RNAse A (Thermo Fisher Scientific) and purified with 1.8X AMPureXP DNA beads. Junction PCR (jPCR) was performed on purified cDNA using core_mCMV_STARRseq_junction forward and reverse primers (Supporting Information Table S1) to specifically enrich reporter transcripts (program: 98°C for 45 s, 16 cycles of [98°C for 15 s, 65°C for 30 s, 72°C for 15 s/30 s], 72°C for 120 s). Extension time was 15 s for targeted approaches ( ~ 500 bp inserts) and 30 s for whole‐genome ( ~ 800 bp inserts). PCR products were purified using 1.8X AMPure beads. To generate Illumina‐compatible sequencing libraries, the purified PCR was used as template for the second PCR (program: 98°C for 45 s, 5 cycles of [98°C for 15 s, 65°C for 30 s, 72°C for s], 72°C for 120 s) with the KAPA HiFi Hot Start Ready Mix (Roche) and NEBNext Multiplex Oligos for Illumina (NEBNext Multiplex Oligos for Illumina, NEB).

#### 2.6.5 Next‐Generation Sequencing

Targeted STARR‐seq libraries for cell harvesting time optimization were sequenced on an Illumina NextSeq sequencer as paired‐end 150 cycle runs. Genome‐wide STARR‐seq libraries were sequenced on an Illumina NextSeq sequencer as paired‐end 75 cycle runs. Targeted STARR‐seq libraries for STARR‐seq peak length optimization were sequenced on an Illumina MiSeq sequencer as paired‐end 75 cycle runs. All sequencing was carried out at the Babraham Genomics Facility.

### 2.7 Targeted STARR‐seq Data Analysis

For the analysis of targeted STARR‐seq data, a custom reference file containing the FASTA sequences for the targeted fragments was used. Alignment was performed with Bowtie version 1.3.1 (bowtie ‐v 3, ‐m 1, ‐‐best, ‐‐strata, ‐‐minins 300, and ‐‐maxins 1000). Coverage for each fragment in both the input and RNA libraries was calculated using GATK version 4.5 CollectHsMetrics (Picard). Reads per target were normalized to reads per million (RPM), and for each fragment, STARR‐seq activity was calculated as the fold change between RPM in the RNA and input libraries.

### 2.8 Whole‐Genome STARR‐seq Data Analysis

Paired‐end sequencing data from the whole‐genome STARR‐seq input and RNA libraries was mapped to the Chinese hamster genome (CriGri‐PICRH‐1.0; NCBI RefSeq assembly: GCF_003668045.3) using Bowtie2 version 2.5.3 (bowtie2 options ‐‐met‐stderr ‐‐maxins 1500 ‐‐no‐mixed). The resulting SAM files were filtered using SAMtools version 1.19.1 (samtools view ‐F 3852 ‐f 2 ‐q 40) to retain only uniquely mapped reads with a mapping quality score greater than 40. Genome coverage BigWig files were generated from filtered reads using DeepTools version 3.5.4 and normalized to reads per kilobase per million mapped reads (RPKM) (bamCoverage ‐‐normalizeUsing RPKM ‐‐extendReads). To assess reproducibility, the 2 biological replicates were processed separately, yielding a high correlation (*R* ^2^ = 0.94). Reads from biological replicates were combined for all subsequent analyzes. Peak calling was performed using STARRPeaker (Lee et al. 2020) with combined STARR‐seq replicates versus input. Only peak calls (*n* = 71,493) with *p* < 10^–5^, an enrichment over input > 2.5 and located within regular chromosomes (1‒10 and X) were kept for further analysis (*n* = 13,395). STARR‐seq peaks were annotated as “accessible” if they overlapped with an ATAC‐seq peak. Peaks were also annotated as H3K27ac+ if they overlapped with a corresponding ChIP‐seq peak and as H3K4me1+ if they fell within a ± 3 kb flanking region of an H3K4me1 ChIP‐seq peak.

### 2.9 Strength‐Based Classification of STARR‐seq Enhancers

Enhancers were classified according to their strength into “strong”, “intermediate”, or “weak” categories, using a method previously described (Barakat et al. 2018). In brief, we ranked STARR‐seq enhancers based on their STARR‐seq activity and defined points of change in the mean and variance of the data using the changepoint package version 2.2.4 in R (Killick and Eckley 2014). The highest value was used as the lower threshold for strong enhancers (STARR‐seq activity (log_2_) = 4.0) and the lowest value as the upper threshold for weak enhancers (STARR‐seq activity (log_2_) = 3.1), enhancers in between these thresholds were classified as intermediate.

### 2.10 ATAC‐seq and ChIP‐seq Data Reanalysis

We used ATAC‐seq data from (Bevan et al. 2021) and ChIP‐seq data for histone modifications from (Feichtinger et al. 2016). Raw FASTQ files for H3K4me1 (Run ERR868153, BioSample SAMEA3373341), H3K27ac (Run ERR868155, BioSample SAMEA3373340), H3K9me3 (Run ERR868152, BioSample SAMEA3373360), and input (Run ERR868150, BioSample SAMEA3373339) samples were downloaded from the Sequence Read Archive (Accession: ERP010370, BioProject: PRJEB9291). Both ATAC‐seq and ChIP‐seq reads were aligned to the Chinese hamster genome (CriGri‐PICRH‐1.0; NCBI RefSeq assembly: GCF_003668045.3) using Bowtie2 version 2.5.3. Peak calling was performed using MACS version 2.2.7.1, applying default parameters. Narrow peak calling flag was used for ATAC‐seq, H3K4me1, and H3K9me3, while broad peak calling flag was used for H3K27me3. All samples were normalized by sequencing depth (in millions of reads) using the ‐SPMR flag. Genome coverage BigWig files were generated with DeepTools version 3.5.4, normalized to RPKM (bamCoverage ‐‐normalizeUsing RPKM ‐‐extendReads). Enrichment heatmaps were generated using DeepTools computeMatrix and plotHeatmap functions.

### 2.11 Genomic Annotation of STARR‐seq Peaks and Nearest Gene Assignment

We used HOMER, version 4.11.1 annotatePeaks.pl function and GCF_003668045.3_CriGri‐PICRH‐1.0.ensGene.2023_01.gtf annotation file to determine the location of STARR‐seq peaks in terms of genomic features, assign them to their putative target gene and calculate the distance to the nearest TSS.

### 2.12 Gene Expression Data

mRNA expression data was generated from the CHOK1SV GS‐KO cells, with sequencing conducted on day 4. The FASTQ files are aligned to PICRH genome using STAR aligner and BAM files are generated. The sequence BAM files are converted to bedGraph file using bedtools genomecov and then bedGraph is converted to BigWig sequence coverage files using bedGraphToBigWig module.

### 2.13 Gene Classification Based on Tissue Distribution

We used data from The Human Protein Atlas (Thul and Lindskog 2018) to classify genes based on their tissue expression patterns. Specifically, we focused on the “RNA tissue specificity” field for each gene. Genes labeled as “low tissue specificity” were classified as “ubiquitous,” indicating broad expression across multiple tissues. Conversely, genes labeled as “tissue‐enhanced,” “tissue‐enriched,” or “group enriched” were classified as “tissue‐specific,” reflecting their predominant or selective expression in particular tissues.

### 2.14 Gene Ontology Pathway Enrichment Analysis

Gene ontology (GO) enrichment analysis was performed using PANTHER 19.0, with the GO Biological Process data set and *Mus musculus* as the reference list. The analysis was done on genes associated with the top accessible (*n* = 300) and masked (*n* = 300) STARR‐seq peaks. For the accessible peaks, genes classified as “tissue‐specific” (*n* = 1,465) were tested for enrichment in a separate analysis. A *p*‐value threshold of 0.05 was applied to determine significance. The fold enrichment of queried genes over the expected frequency is presented, alongside the False Discovery Rate (FDR) calculated using the Benjamini‐Hochberg procedure.

### 2.15 Transcription Factor Motif Enrichment Analysis and Scanning

We used the findMotifs.pl function from HOMER version 4.11.1 to identify TF binding motifs that were differentially enriched in enhancer sequences compared to background genomic sequences. To generate a set of background genomic sequences with a distribution comparable to that of STARR‐seq peaks, we took the genomic coordinates of unfiltered STARR‐seq peak calls and shifted them 10 kb downstream (*n* = 71,493). Background regions that overlapped with a STARR‐seq peak or had poor coverage in the input library were filtered out, resulting in a final set of background sequences (*n* = 63,960). To identify TF motifs enriched in strong versus weak STARR‐seq enhancers, we used strong (STARR‐seq activity (log_2_) = 4.0) STARR‐seq enhancers as the target regions and weak (STARR‐seq activity (log_2_) = 3.1) enhancers as the background regions. Separate analyzes were conducted for accessible and closed STARR‐seq enhancers. For motif scanning, we used MEME Suite version 5.5 and FIMO function with default settings to find all motif instances (*p* < 10^–4^) of specific binding motifs.

### 2.16 YY1 Binding Motif Mutagenesis

For the top 50 STARR‐seq peaks containing two or more YY1 binding motif instances (*p* < 10^–4^), we designed shorter sequence versions encompassing all YY1 motif instances. These sequences were *in silico* mutated by replacing the four conserved nucleotides within the YY1 binding motif “ATGG” with “CTCG.” Both wild‐type and mutant sequences were synthesized as gene fragments (Twist Bioscience) and subsequently tested to quantify their STARR‐seq activity.

### 2.17 DeepSTARR Training and Interpretation

Scripts from de Almeida et al. (2022) were used to train and interpret the DeepSTARR model. To prepare the data used for training and evaluating our model, STARR‐seq peaks were binned into 249 bp windows centered on the peak summit, with a stride of 100 bp. For each peak, the central window and 3 additional windows on either side were selected. Additionally, we incorporated a diversity of 249 bp background sequences. In total, this process generated 154,824 sequences that were randomly split into the training (80%), validation (10%), and testing (10%) datasets. To avoid any data leakage, all windows originating from the same peak were kept within the same data set. STARR‐seq activity for each bin was calculated as the fold change between the number of reads overlapping the window in the RNA library and the corresponding number of reads in the input library. Sequences in the training and validation datasets, along with their calculated STARR‐seq activities, were used as input for training the DeepSTARR model. The performance of the model was evaluated by comparing the predicted activity values against the experimentally measured activity values for the sequences in the test data set.

## 3 Results

### 3.1 Systematic Identification of Genomic Regions With Enhancer Activity in CHO Cells

**Figure 1:**
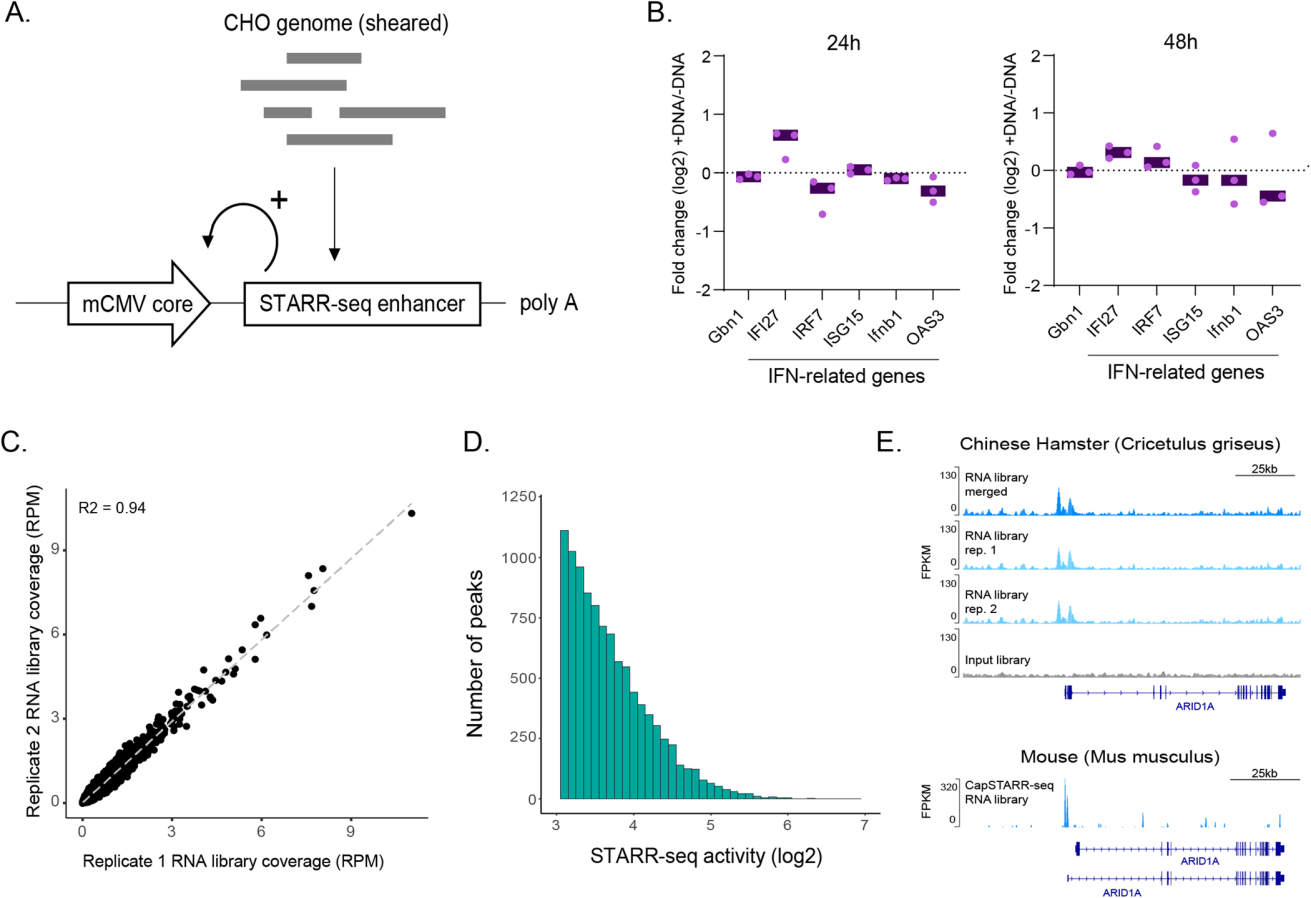
Whole‐genome STARR‐seq in CHO cells. (A) Schematic representation of the whole‐genome STARR‐seq approach using an mCMV core as a promoter for the STARR‐seq screening plasmid. (B) qPCR‐based assessment of ISG‐mRNA induction 24 and 48 h after DNA transfection into CHO cells. Individual data points shown for three independent replicates. A non‐parametric Wilcoxon paired test was used to assess for significant differences between *Gnb1* housekeeping gene and interferon response‐related genes; no significant differences were observed. (C) Correlation of STARR‐seq RNA library coverage for two biological replicates. Shown are fragment counts for 500 bp genomic windows, normalized to reads per million. (D) Histogram depicting STARR‐seq activity (enrichment of STARR‐seq RNA over input library) for 13,395 whole‐genome STARR‐seq peaks ranked from lowest to highest. (E) Coverage profiles for STARR‐seq RNA libraries in CHO (replicates 1 and 2, and the merged reads from both replicates) and mouse are displayed in blue, while the input library is shown in gray for the *ARID1A* locus. The top panel represents whole‐genome STARR‐seq data from CHO cells, and the bottom panel shows Cap‐STARR‐seq for 3T3 mouse cells (Vanhille et al. 2015). A common STARR‐seq enhancer detected by both techniques and conserved across both species is shown. RPKM, reads per kilobase per million mapped reads.

To identify genomic regions with enhancer activity in CHO cells, we conducted whole‐genome STARR‐seq. A genome‐wide plasmid library was made by cloning randomly sheared genomic fragments (average size ~ 800 bp) into a STARR‐seq expression vector at a site downstream of a core mCMV promoter (Figure 1A). We replaced the standard STARR‐seq Ori with the mCMV core promoter for two reasons: (i) The mCMV core promoter is well‐characterized and known to function as a core promoter in CHO cell lines (Chatellard et al. 2007), and (ii) performing STARR‐seq using this sequence would directly highlight regions of the CHO genome capable of exerting enhancer activity upon this proximal promoter sequence, thereby suggesting enhancer‐core (mCMV) promoter sequence combinations that may be repurposed within recombinant protein expression vector designs with potentially minimal further modifications. The proportion of the genome captured in the STARR‐seq plasmid library was quantified by high‐throughput sequencing of genomic DNA inserts. The mapped fragments covered 96% of the genome, with 86% of the genome covered at least 5X, indicating good genome representation.

We next optimized several steps of the STARR‐seq assay. First, as DNA transfection can trigger an interferon response in certain cell types and such responses can confound enhancer activity assays (Muerdter et al. 2018), we investigated whether a similar response occurs in CHO cells. We measured the expression of IFN‐related genes in transfected and non‐transfected cells at 24 h and 48 h posttransfection. Our analysis showed no significant differences in the expression ratios (transfected over non‐transfected) of IFN‐related genes compared to the housekeeping gene *Gnb1*, whose expression is expected to remain stable across conditions (Figure 1B). This result indicates that PEI‐based DNA transfection does not provoke a detectable IFN response in CHO cells. Second, we optimized the timing of when to measure enhancer activity following plasmid transfection, selecting 6 h and 24 h timepoints that have been used in prior studies (Barakat et al. 2018; Neumayr et al. 2019). As expected, there was a strong correlation between the two time points (*R* ^2^ = 0.89; Supporting Information Figure S3A). However, the signal‐to‐noise ratio was significantly higher at 6 h, indicating that earlier sampling provides more reliable enhancer activity quantification (Supporting Information Figure S3B). When comparing enhancer activity levels measured using the Ori‐based vector (Muerdter et al. 2018) with those obtained using the mCMV core promoter vector, we observed a high degree of correlation (*R* ^2^ = 0.85 at 6 h and *R* ^2^ = 0.86 at 24 h posttransfection; Supporting Information Figure S4). This consistency across the two vectors confirms the reliability of the mCMV‐driven vector for accurate quantification of enhancer activity.

CHO cells were transfected with the whole‐genome STARR‐seq plasmid library and harvested 6 h later. To assess the enhancer activity of each genomic fragment, we quantified the transcripts originating from the core mCMV promoter by RNA‐sequencing. The RNA coverage profiles were highly concordant between replicates (*R* ^2^ = 0.94; Figure 1C), and hence we pooled replicates for all subsequent analyzes. By comparing the number of expressed fragments to their abundance in the input library, we identified 13,395 regions with enhancer activity (defined as log_2_ fold‐change ≥ 2.5 and *p* < 10^–5^). These STARR‐seq active genomic regions (hereafter STARR‐seq enhancers) have an input‐corrected enrichment ranging from sixfold to 117‐fold (Figure 1D; Supporting Information Table S3). Notably, among the identified enhancers, we observed sequences homologous to regions in the mouse genome that have known STARR‐seq activity (Vanhille et al. 2015), thereby validating our approach (Figure 1E). Together, these results provide a systematic assessment of regions in the CHO cell genome that have enhancer activity.

### 3.2 Using Newly Identified STARR‐seq Enhancers for Recombinant Protein Expression

**Figure 2:**
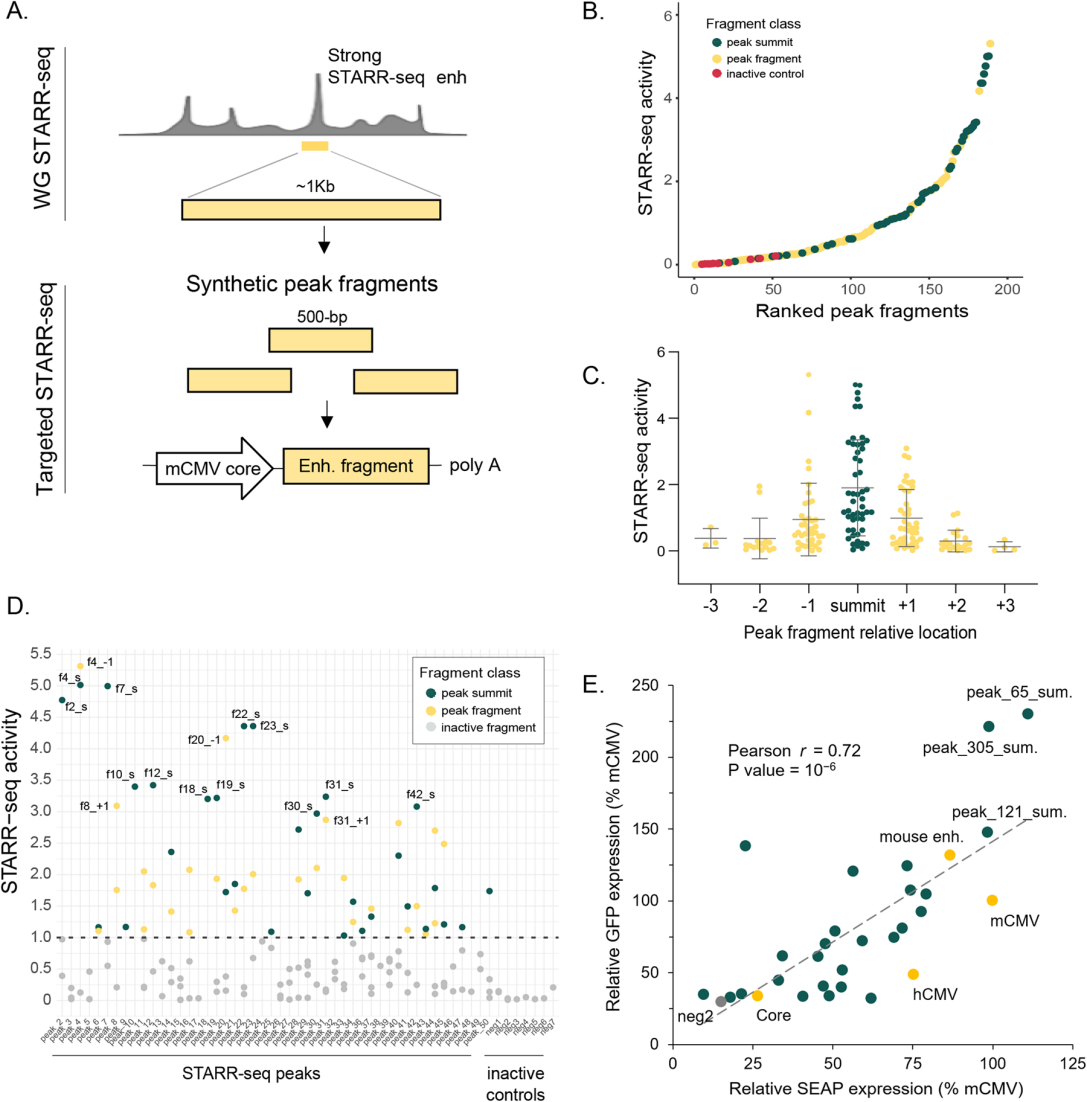
Newly identified STARR‐seq enhancers for recombinant protein expression. (A) Design of the targeted STARR‐seq experiment. (B) STARR‐seq activity of enhancer fragments screened in the targeted experiment. Green dots represent fragments overlapping a whole‐genome peak summit; yellow dots represent fragments overlapping other peak regions; red dots represent inactive control regions. (C) Average STARR‐seq activity of peak fragments overlapping the original peak summit (labeled as “summit”) and the consecutive upstream (‒1, ‒2, ‒3) and downstream ( + 1, + 2, + 3) contiguous fragments. Boxes are interquartile range; line is mean; whiskers are standard deviation. (D) STARR‐seq activity for 500 bp peak fragments corresponding to the top 50 most active peaks in the whole‐genome STARR‐seq experiment and control inactive regions. Fragments with no STARR‐seq activity (FC < 1) are in gray. Active fragments (FC > 1) overlapping the original peak summit are depicted in blue, while summit‐adjacent fragments are shown in yellow. The fragment ID is indicated for those fragments tested in the GFP experiment. (E) Scatter plot showing GFP and SEAP protein expression levels for various STARR‐seq enhancers (Supporting Information Table S5), normalized to the full‐length mCMV promoter/enhancer (mCMV). The hCMV promoter/enhancer and a previously described enhancer from the mouse genome (Vanhille et al. 2015; Mouse enhancer), which has a homologous sequence in the CHO genome, were included as positive controls.

To improve STARR‐seq enhancer length resolution for the strongest sequences and to optimize their size for compatibility with recombinant protein expression platforms, we carried out a targeted, secondary screen. The top 50 active regions identified in the genome‐wide STARR‐seq data set were *in silico* fragmented into 500 bp regions with 200 bp overlap with neighboring fragments. We synthesized these 500 bp fragments and tested 159 of them using STARR‐seq (Figure 2A; Supporting Information Table S4). The average activity of fragments overlapping the summit of a STARR‐seq enhancer was higher compared to flanking fragments (Figure 2B,C). Of the STARR‐seq enhancers tested in the secondary screen, 75% had at least one smaller fragment with enhancer activity, whereas none of the inactive regions showed activity (Figure 2D). These results indicate that 500 bp is sufficient to capture enhancer activity for most sequences tested.

Next, we assessed the ability of length‐optimized STARR‐seq enhancer sequences to stimulate the expression of recombinant proteins when located upstream of the core mCMV promoter. We selected the most active enhancer fragments (*n* = 16) along with 10 additional 500 bp fragments centered on whole‐genome STARR‐seq peak summits that had not been tested in the initial targeted experiment. These sequences were cloned into GFP and SEAP reporter plasmids, and the resulting protein expression levels were normalized to that of the full‐length mCMV promoter/enhancer, a strong viral regulatory element (Figure 2E; Supporting Information Table S5). There was a moderate but highly significant correlation between GFP and SEAP expression (Pearson correlation coefficient = 0.72, *p* = 10^‐6^). Differences between GFP and SEAP signals were likely due to differences in reporter characteristics (e.g., intracellular turnover of GFP, extracellular secretion and accumulation of SEAP), and potential saturation of SEAP expression under the transient transfection conditions (Johari et al. 2015). 22 out of 26 sequences (85%) drove both GFP and SEAP expression levels above those of the core‐only plasmid, confirming their functional enhancer activity. Additionally, the top three sequences showed significantly higher GFP expression than the mCMV control (≳ 150%; *p* < 0.01), implying highly active enhancers.

### 3.3 Chromatin Context of STARR‐seq Enhancers

**Figure 3:**
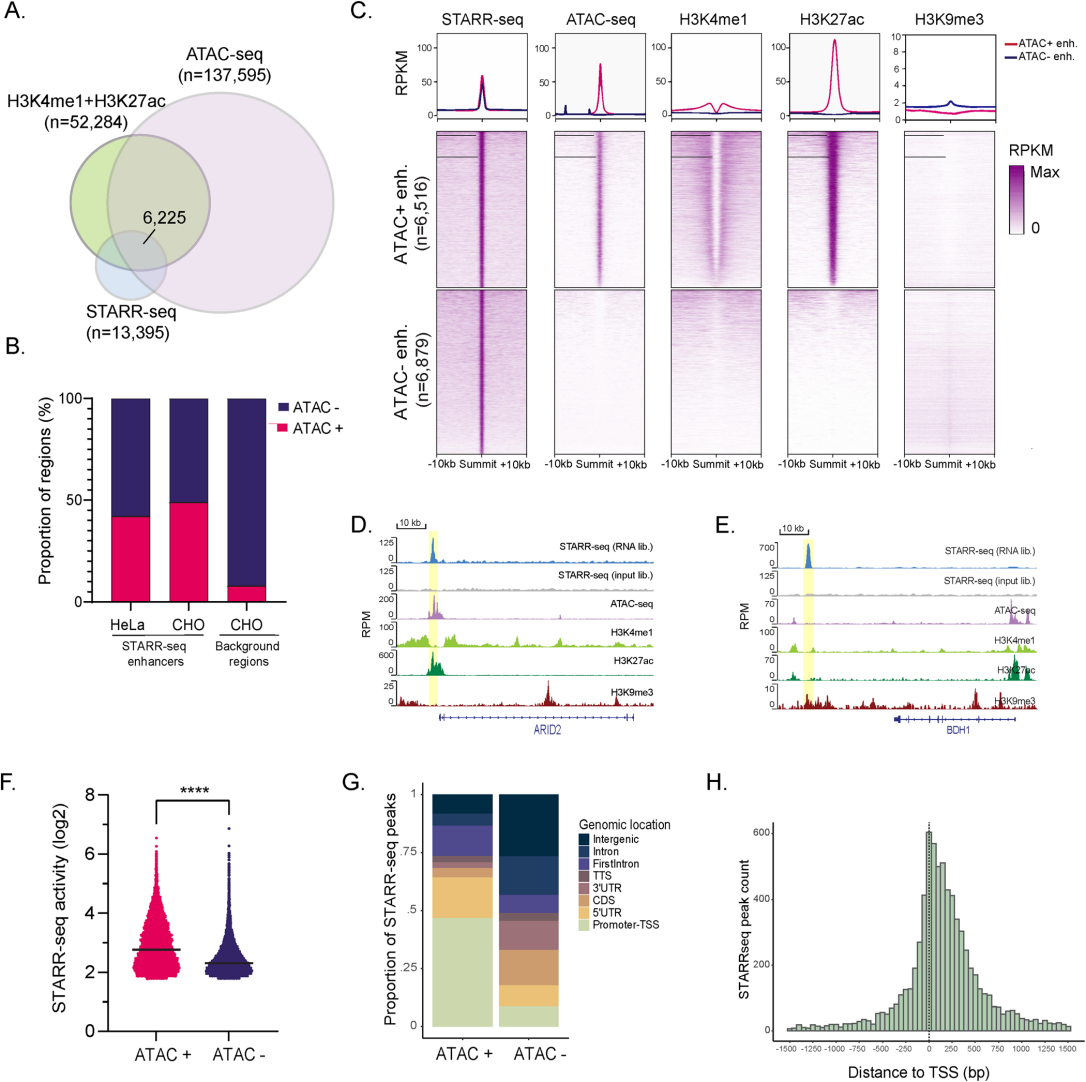
Chromatin context of STARR‐seq enhancers. (A) Venn diagram showing the overlap between H3K4me1 and H3K27ac, ATAC‐seq and STARR‐seq peaks. The number of peaks in each group and the overlap among the three datasets are indicated. (B) Proportion of STARR‐seq enhancers and background genomic regions overlapping accessible (ATAC +) or non‐accessible (ATAC ‒) chromatin in HeLa (Muerdter et al. 2018) and CHO cells. (C) Average coverage (top) and coverage heatmaps (bottom) of STARR‐seq, ATAC‐seq, H3K4me1, H3K27ac, and H3K9me3 ChIP‐seq libraries across STARR‐seq enhancers in chromatin accessible (ATAC + enh.) and non‐accessible (ATAC ‒ enh.) regions. STARR‐seq (RNA and input libraries) and chromatin profiles at representative loci, illustrating (D) accessible and (E) masked STARR‐seq enhancers. (F) STARR‐seq activity (enrichment of STARR‐seq RNA over input library) for accessible (ATAC + ) or non‐accessible (ATAC ‒) STARR‐seq enhancers. Line represents median activity. Two‐sided unpaired *t*‐test. (G) Stacked charts show the proportion of different genomic annotations for STARR‐seq enhancers. (H) Histogram illustrating the average distances from STARR‐seq enhancers to the nearest TSS. The dashed line represents the location of the TSS. CDS, coding sequence; TSS, transcription start site; TTS, transcription termination site; UTR, untranslated region.

To investigate the status of STARR‐seq enhancers in their endogenous chromatin context, we integrated STARR‐seq with chromatin accessibility (Bevan et al. 2021) and histone modification data sets (Feichtinger et al. 2016). Interestingly, only 16% (*n* = 6,225) of genomic sequences annotated as putative active enhancers (H3K4me1 + ; H3K27ac + ; ATAC‐seq + ) overlapped with STARR‐seq enhancers (Figure 3A). This finding is consistent with previous reports (Bozek and Gompel 2020) and indicates that chromatin‐based signatures identify a broad class of regulatory elements of which only a subset have enhancer activity. Overall, 49% (*n *= 6,516) of STARR‐seq enhancers overlapped accessible chromatin regions (ATAC‐seq +), a percentage comparable to that reported for STARR‐seq in other cell types (Muerdter et al. 2018) (Figure 3B). Most of the STARR‐seq enhancers within this group were enriched for enhancer‐associated histone modifications in CHO cells (Figure 3C,D). In contrast, STARR‐seq enhancers corresponding to regions in inaccessible chromatin (ATAC‐seq –) did not show enrichment for H3K4me1 or H3K27ac but did show a very weak enrichment for the repressive histone modification H3K9me3 (Figure 3C,E). These sites likely correspond to regions with potential enhancer activity that are masked or silenced through heterochromatin‐associated pathways (hereafter termed masked enhancers).

We observed higher STARR‐seq activity for STARR‐seq enhancers within accessible chromatin compared to those within inaccessible chromatin (Figure 3F). Interestingly, however, several masked enhancers were highly active when taken out of their endogenous context and measured by STARR‐seq, including sequences in the loci of *ADGRB1* (a regulator of dendritic spine development) and *CAMKII* (a gene with a strong constitutive expression in mouse brain), suggesting chromatin‐mediated silencing of strong enhancers to prevent the expression of their target gene in inappropriate cell types, such as CHO cells. These regions, nevertheless, may still be useful in ectopic systems to drive high levels of transgene expression.

Regarding the genomic distribution of accessible and masked enhancers (Figure 3G), about half of endogenously active enhancers overlapped with or were proximal to a TSS (TSS‐proximal enhancers), being preferentially located from + 1 to + 500 bp downstream of the TSS (Figure 3H). By contrast, masked enhancers were mostly located in intergenic (27%) or intronic (25%) regions (TSS‐distal enhancers) attending to a more canonical definition of enhancers.

### 3.4 Functional Differences of Genes Associated With Accessible and Masked STARR‐seq Enhancers

**Figure 4:**
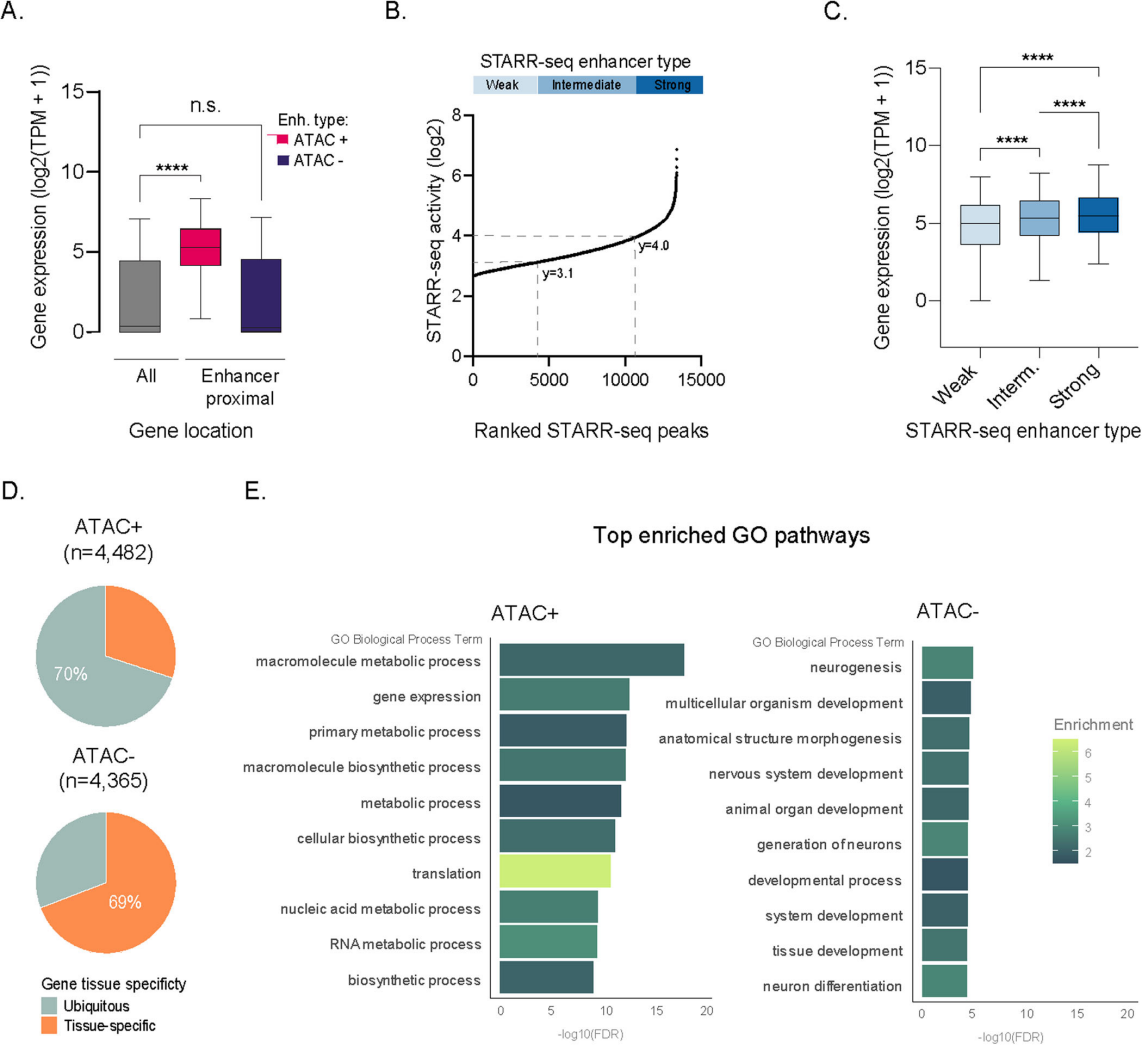
Expression of genes associated with STARR‐seq enhancers. (A) Boxplot of gene expression for all genes (All), genes proximal to accessible enhancers (ATAC + ), and genes proximal to repressed enhancers (ATAC ‒ ). Boxes show median with interquartile range; whisker show the 5th and 95th percentiles. Adjusted *p*‐value from one‐way ANOVA. (B) Plot showing ranked STARR‐seq enhancer activity. Enhancers were classified as weak, intermediate or strong based on their activity. Dashed lines indicate the activity thresholds. (C) Boxplot of expression levels for genes proximal to weak, intermediate, and strong enhancers. Boxes show median with interquartile range; whisker show the 5th and 95th percentiles. Adjusted *p*‐value from one‐way ANOVA. TPM, transcript per million; ****p < 0.0001; n.s., nonsignificant. (D) Pie charts depict tissue expression patterns from the Human Protein Atlas of genes near accessible (top) and masked (bottom) STARR‐seq enhancers. (E) Bar graph presenting the top 10 most significantly enriched GO terms for genes near the top 300 accessible (ATAC + ) and masked (ATAC ‒) enhancers. The graph shows the minus log_10_‐transformed false discovery rate (FDR)‐adjusted *p*‐values (Fisher's exact test) and fold enrichment.

To investigate the relationship between STARR‐seq enhancers and gene expression in CHO cells, we assigned each enhancer to a putative target gene based on genomic proximity. Genes located near to accessible STARR‐seq enhancers showed higher than average gene expression levels in CHO cells (Figure 4A) with expression levels correlating with intrinsic enhancer strength (Figure 4B,C). In contrast, genes associated with masked enhancers were expressed at levels similar to the overall average.

Genes linked to accessible and masked enhancers revealed different tissue expression patterns (Figure 4D). ATAC‐seq + enhancers are predominantly located near genes with ubiquitous expression, including housekeeping and ribosomal protein genes, whereas ATAC‐seq ‒ enhancers were preferentially associated to genes with more restricted, tissue‐specific expression. GO pathway enrichment analysis further underscored the distinct roles of these enhancer types. Genes associated with accessible enhancers were enriched in pathways related to metabolic and core cellular processes, such as gene expression or translation (Figure 4E). In contrast, genes associated with masked enhancers were more often involved in developmental pathways and pathways specific to non‐ovarian tissue. Of note, the subset of tissue‐specific genes near accessible enhancers was enriched in pathways related to CHO cell identity, including epithelial cell development (GO:0030855, 1.74‐fold enrichment, FDR = 6.42 × 10^‒4^) and reproductive system development (GO:0061458, 1.8‐fold enrichment, FDR = 0.03). These findings suggest that while most active enhancers are linked to core cellular functions, a minority are involved in cell‐specific processes, potentially contributing to tissue identity.

### 3.5 The Transcription Factors YY1 and ETS are Associated With Strong Enhancer Activity

**Figure 5:**
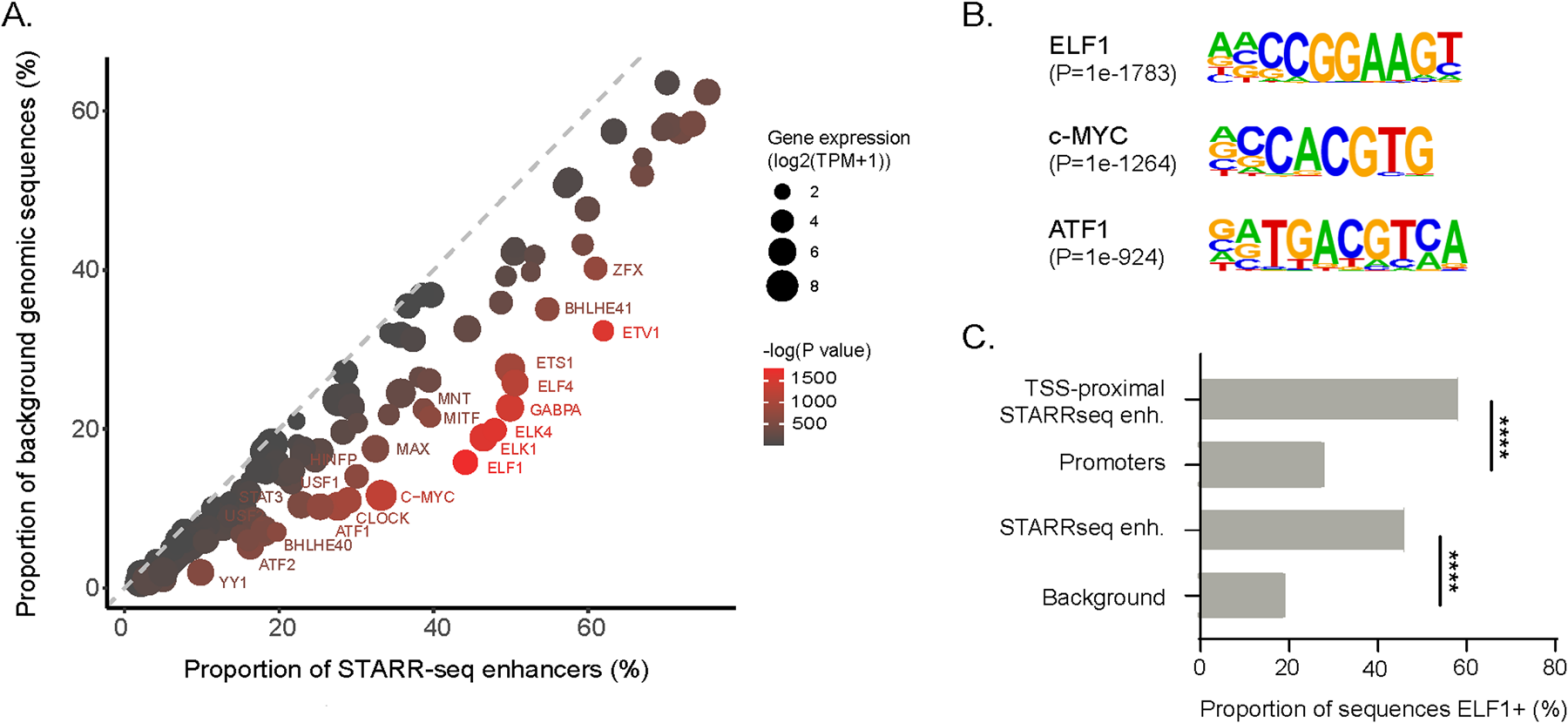
Transcription factors (TFs) mediating enhancer activity in CHO. (A) Scatter plot illustrating the percentage of sequences harboring TF binding motifs significantly enriched in STARR‐seq enhancers compared to background sequences. The size of the dots is proportional to the expression levels of the specific TF in CHO cells. Dot color represents the *p*‐value. (B) Top 3 nonredundant enriched TF binding motifs in STARR‐seq enhancers over background regions. The *p*‐value for each enrichment is indicated in brackets. (C) Bar graph showing the proportion of ETS‐family member ELF1 motifs in TSS‐proximal STARR‐seq enhancers (TSS‐proximal STARRseq enh.), promoter regions not overlapping STARR‐seq peaks in the CHO genome (Promoters), STARR‐seq enhancers (STARRseq enh.) and background genomic regions (Background).

To identify TFs that may mediate enhancer function in CHO cells, we compared the occurrence of TF binding motifs in STARR‐seq enhancers to background genomic regions. This analysis revealed a significant enrichment of motifs for broadly expressed TFs and ubiquitous activators, including ETS family members and ATF1/2 (Figure 5A,B). Binding motifs for ETS family members were particularly enriched in TSS‐proximal enhancers compared to other promoter regions in the genome (Figure 5C). We also observed significant enrichment of motifs for other TFs including c‐MYC, YY1, E2F1, CHOP, CEBP, and ATF4.

**Figure 6:**
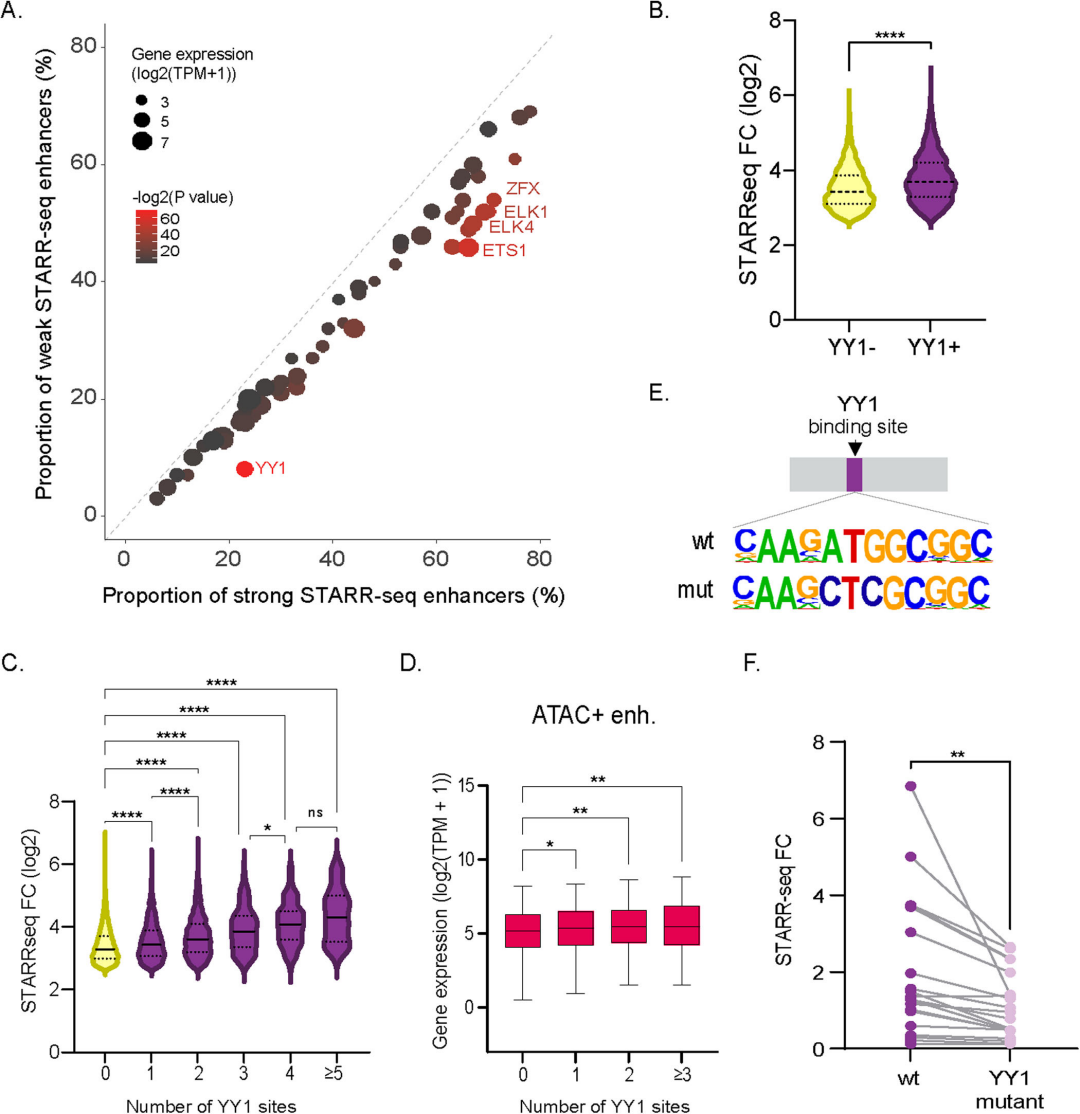
YY1 is associated with enhancer strength. (A) Scatter plot showing transcription factor (TF) binding motifs enriched in strong over weak STARR‐seq enhancers. Dot size is proportional to the expression levels of specific TFs in CHO cells. Dot color represents the *p* value, ranging from brown (low significance) to red (high significance). (B) STARR‐seq activity for enhancers with and without YY1 binding motifs. The central line represents the median activity, and the top and bottom dashed lines represent the quartiles. Two‐sided unpaired *t*‐test. (C) STARR‐seq activity for enhancers based on the number of YY1 binding motifs, ranging from 0 to 5 or more. Adjusted *p*‐values from one‐way ANOVA. (D) mRNA expression levels for genes located near STARR‐seq enhancers in accessible chromatin, categorized by the number of YY1 binding motifs. (E) Schematic representation of wild‐type (wt) YY1 binding motifs and the mutated motif version with two bases altered. (F) STARR‐seq activity for wild‐type enhancers compared to their mutated versions, where all YY1 binding motifs were replaced by mutant sequences. Statistical significance is indicated as follows: ****p < 0.0001; **p < 0.01; *p < 0.05; n.s., nonsignificant.

To identify TFs that drive the highest levels of gene expression in CHO cells, we compared motif enrichment between strong and weak accessible STARR‐seq enhancers. This analysis revealed a significant enrichment of the YY1 motif (*p* = 10^–69^), with 23% of strong enhancers containing this motif—a value that is double the proportion in weak enhancers and > 20 times that of background regions (Figure 6A). To investigate the sequence determinants of enhancer strength using an orthogonal approach, we applied the DeepSTARR deep learning model (de Almeida et al. 2022). DeepSTARR is designed to run on any large set of DNA sequences with corresponding STARR‐seq activity measures. The model can predict enhancer activity from previously unseen DNA sequences and assess the contribution of individual nucleotides to the overall sequence activity. We trained the model using whole‐genome STARR‐seq data from CHO cells, achieving a Pearson correlation coefficient of 0.77 between predicted and observed enhancer activity on a test data set (Supporting Information Figure S5A). This coefficient value is comparable to the value reported in the study that developed DeepSTARR (de Almeida et al. 2022) and indicates that the model accurately captures regulatory information. DeepSTARR analysis of sequences with the highest activity identified YY1 and ETS binding motifs as the major contributors to enhancer activity (Supporting Information Figure S5B). Further analysis showed that STARR‐seq enhancer activity was significantly higher for regions containing at least one YY1 binding site compared to those without YY1 sites (Figure 6B). The number of YY1 motifs within an enhancer correlated linearly with activity, with sequences containing 5 or more YY1 motifs exhibiting the highest enhancer activity (Figure 6C). There was no difference on average ATAC‐seq signals for sequences with different numbers of YY1 motifs. Additionally, the expression level of genes adjacent to accessible enhancers was positively correlated with the number of YY1 motifs (Figure 6D), whereas no significant difference was observed for masked enhancers.

Lastly, to investigate the importance of YY1 for enhancer activity, we measured STARR‐seq activity for 25 enhancer regions containing either wild‐type sequences or matched sequences with mutant YY1 motifs. We mutated the YY1 motifs by altering two bases, converting the conserved ATGG sequence into CTCG, which is known to disrupt YY1 binding (Wu and Lee 2001) (Figure 6E). This mutation led to a substantial reduction in enhancer activity, particularly for the strongest activities, with up to a fivefold decrease (Figure 6F). Overall, these data support a role for YY1 as a key mediator of strong enhancer activity in CHO cells.

## 4 Discussion

Optimizing transgene expression in CHO cells is important for improving therapeutic protein production, particularly of the latest generation of multi‐chain biotherapeutics. However, strategies to achieve this have been partly constrained by the limited knowledge of cis‐ and trans‐regulators of gene expression in this critical cell type. This knowledge gap has hindered efforts to effectively engineer both the host cell lines themselves as well as the associated expression vectors for enhanced and stable protein production, thereby potentially slowing progress in biotherapeutic manufacturing. By using high‐throughput methods to quantify reporter gene expression, together with epigenome profiles, our study provides a comprehensive map of regions with enhancer activity across the CHO cell genome. Unlike previous approaches that relied solely on chromatin states for predicting regulatory elements (Bevan et al. 2021; Feichtinger et al. 2016; Lee et al. 2021), our STARR‐seq data provide direct assessment of the ability of enhancer regions to induce transcription. As a result, this study provides a repository of empirically‐determined CHO enhancer ‘building blocks’ covering a wide dynamic range of enhancer activity, that could potentially be used to augment current expression vector designs for recombinant protein expression, or achieve specific ‘tuneable’ levels of transgene expression.

Integration of STARR‐seq data with epigenome profiles revealed that a substantial proportion of sequences with ectopic enhancer activity are masked by inaccessible chromatin in their endogenous loci. This finding is consistent with prior reports in *Drosophila* and human cells (Muerdter et al. 2018). Also consistent with previous findings (Lee et al. 2021), masked enhancers were associated with tissue‐specific pathways, including neuronal‐related pathways. These masked sequences are overlooked when relying only on epigenomics‐based approaches to predict genomic enhancers, yet they provide attractive examples to study the mechanisms of chromatin‐mediated repression, and underscore the value of using methods that read out enhancer activity directly.

Active enhancers identified in CHO cells were predominantly TSS‐proximal and frequently overlapped with promoters of housekeeping and other ubiquitously expressed genes. This finding is in line with studies in *Drosophila* cells (Zabidi et al. 2015) which demonstrated that promoters of housekeeping genes often function as enhancers. Additionally, Dao and colleagues reported that approximately 2% of promoters in mammalian cells exhibit enhancer activity. These promoters are typically associated with genes involved in fundamental cellular processes and tend to be less tissue‐specific than distal enhancers (Dao et al. 2017). Housekeeping genes, which are essential for normal cell metabolism and growth, require precise coordination to adapt to the cell's dynamic metabolic demands. However, their promoters often lack interactions with distal enhancers, and genomic regions densely packed with housekeeping genes usually contain few distal enhancers (Zhu et al. 2021). Evidence suggests that housekeeping genes may coordinate their expression through interactions between their promoters (Dejosez et al. 2023). Indeed, TSS‐proximal enhancers have been shown to function as bona fide enhancers and can regulate the expression of distal genes within their native context (Dao et al. 2017). In CHO cells, further research is necessary to understand how these enhancers regulate gene expression in their native genomic environment.

Measuring enhancer activity genome‐wide enabled us to determine potential mediators of gene regulation in CHO cells. Our results identified an enrichment in STARR‐seq enhancers of binding motifs for ETS transcriptional activators, such as ELF1, ELK1, ELK4, and GABPA, and the presence of these motifs was positively correlated with enhancer strength. These findings are relevant for informing the choice of TF overexpression approaches, which is a potentially viable strategy to enhance therapeutic protein production. Indeed, several TFs that are predicted in our study to mediate enhancer activity, including c‐MYC, YY1, E2F1, CHOP, or ATF4, have been overexpressed in CHO cells and led to improved recombinant protein yields (Haredy et al. 2013; Latorre et al. 2023; Majors et al. 2008; Nishimiya et al. 2013; Tastanova et al. 2016). These findings support a role for these TFs in gene regulation in CHO cells. Importantly, our data on enhancer activity provide a likely explanation for why the overexpression of these factors led to higher gene induction, as well as identifying other candidate factors that have not yet been tested. A future research direction could investigate whether the overexpression of ETS factors, either alone or in combination, can elevate transgene yields.

Another important outcome of this study is the identification of YY1 as a trans‐activator in CHO cells. Our results indicate that YY1 binding sites play a crucial role in enhancer activity, particularly for the strongest enhancers, as mutations in these sites led to reduced activity. The number of binding motifs was positively correlated with enhancer activity in an additive manner and this relationship was independent of chromatin accessibility. This suggests that the cooperative effect of YY1 is mediated by mechanisms other than enhancing chromatin accessibility. YY1 is a zinc finger TF that functions as both an activator and repressor, with significant roles in gene regulation (Bérubé‐Simard et al. 2014; Policarpi et al. 2024). Notably, YY1 forms dimers that facilitate enhancer‐promoter interactions, crucial for gene expression. Deletion of YY1 binding sites or depletion of YY1 protein disrupts enhancer‐promoter looping and gene expression (Weintraub et al. 2017). Interestingly, CMV promoters contain YY1 binding motifs, where YY1 was proposed to act as a repressor to favor latent infection (Brown et al. 2015; Johari et al. 2022). However, overexpression of YY1 in CHO cells led to a global reprogramming effect and increased gene expression from various endogenous promoters (Tastanova et al. 2016), which aligns with our findings associating YY1 with enhancer activity.

A potential limitation of this study is that STARR‐seq was used to capture the activity of enhancers only during the early phase of CHO cell culture. Many genes are downregulated during the stationary phase or under hypothermic conditions of CHO cell culture, while others, such as apoptosis‐related genes, are upregulated at later stages of culture or under adverse conditions such as stress or nutrient deprivation (Johari et al. 2019). Changes in gene expression occurring at later stages of CHO cell culture may not have been captured in our study, which could leave gaps in understanding enhancer activity during these critical periods. Additionally, although endogenous sequences are functional when used within an episomal context, as shown in this study, it will be important to determine in future studies how these sequences function once reintegrated back into the CHO genome in the context of stable cell lines. It is also worth emphasizing that the genomes of CHO‐derived cell lines have likely undergone extensive rearrangements over their continued growth, and even clonal populations develop into heterogeneous subpopulations very quickly (Wurm and Wurm 2021). Consequently, the functional enhancer mapping presented in this study may be specific to the CHOK1SV GS‐KO® cell line, and further analyzes of other CHO cell lines would be beneficial.

Taken together, this study demonstrates that enhancers can be identified from the genome of an industrially‐relevant CHO cell line and used in an episomal context to drive expression of recombinant proteins such as GFP and SEAP. The most active sequences could activate transgene expression at levels similar to or higher than strong viral enhancers. This study therefore shows that the host cell line itself can be a source of useful sequences to potentially further optimize the expression of recombinant proteins with future benefits for enhanced biopharmaceutical production.

## Author Contributions

**María Santos:** conceptualization, data curation, formal analysis, investigation, methodology, validation, visualization, writing – original draft. **Yusuf Johari:** conceptualization, formal analysis, investigation, methodology, validation, writing – review and editing. **Laura Biggins:** formal analysis. **Natalie Elliott:** investigation. **Stefan Schoenfelder:** methodology. **Mounika Boddireddy:** data curation, formal analysis. **Daniel Fabian:** data curation, formal analysis. **Peter O'Callaghan:** conceptualization, methodology, writing – review and editing. **Michael Anbar:** project administration, supervision. **Peter Rugg‐Gunn:** conceptualization, methodology, supervision, writing – review and editing.

## Conflicts of Interest

Authors MS, YBJ, PO'C, and PR‐G have a patent application filed based on the work in this article.

## Supporting information


**Figure S1:** Schematic representation of maxGFP reporter vectors. **Figure S2:** Schematic representation of mCMV and Ori STARR‐seq vectors. **Figure S3:** Comparison in STARR‐seq activity between samples collected at 6 hr and 24 hr post‐transfection. **Figure S4:** Comparison in STARR‐seq activity quantification between mCMV and Ori as core promoters. **Figure S5:** DeepSTARR model performance and nucleotide contribution prediction.

**Supplementary Table S1:** Primer list. **Supplementary Table S2:** Synthetic gene fragments for cell harvesting time optimization. **Supplementary Table S3:** WG STARR‐seq enhancers. **Supplementary Table S4:** WG STARR‐seq enhancers. **Supplementary Table S5:** Relative GFP and SEAP expression levels of synthetic gene fragments compared to mCMV.

## Data Availability

The data that supports the findings of this study are available in the supporting information of this article.

## References

[bit70076-bib-0001] de Almeida, B. P. , F. Reiter , M. Pagani , and A. Stark . 2022. “DeepSTARR Predicts Enhancer Activity From DNA Sequence and Enables the De Novo Design of Synthetic Enhancers.” Nature Genetics 54, no. 5: 613–624. 10.1038/s41588-022-01048-5. 35551305

[bit70076-bib-0002] Arnold, C. D. , D. Gerlach , C. Stelzer , Ł. M. Boryń , M. Rath , and A. Stark . 2013. “Genome‐Wide Quantitative Enhancer Activity Maps Identified by STARR‐Seq.” Science 339, no. 6123: 1074–1077. 10.1126/science.1232542. 23328393

[bit70076-bib-0003] Barakat, T. S. , F. Halbritter , M. Zhang , et al. 2018. “Functional Dissection of the Enhancer Repertoire in Human Embryonic Stem Cells.” Cell Stem Cell 23, no. 2: 276–288.e8. 10.1016/j.stem.2018.06.014. 30033119 PMC6084406

[bit70076-bib-0004] Bebbington, C. R. 1991. “Expression of Antibody Genes in Nonlymphoid Mammalian Cells.” Methods 2, no. 2: 136–145. 10.1016/S1046-2023(05)80214-2.

[bit70076-bib-0005] Bérubé‐Simard, F. A. , C. Prudhomme , and L. Jeannotte . 2014. “YY1 Acts as a Transcriptional Activator of Hoxa5 Gene Expression in Mouse Organogenesis.” PLoS One 9, no. 4: e93989. 10.1371/journal.pone.0093989. 24705708 PMC3976385

[bit70076-bib-0006] Bevan, S. , S. Schoenfelder , R. J. Young , et al. 2021. “High‐Resolution Three‐Dimensional Chromatin Profiling of the Chinese Hamster Ovary Cell Genome.” Biotechnology and Bioengineering 118, no. 2: 784–796. 10.1002/bit.27607. 33095445 PMC7894165

[bit70076-bib-0007] Boshart, M. , F. Weber , G. Jahn , K. Dorsch‐Hler , B. Fleckenstein , and W. Schaffner . 1985. “A Very Strong Enhancer is Located Upstream of an Immediate Early Gene of Human Cytomegalovirus.” Cell 41, no. 2: 521–530. 10.1016/S0092-8674(85)80025-8. 2985280

[bit70076-bib-0008] Bozek, M. , and N. Gompel . 2020. “Developmental Transcriptional Enhancers: A Subtle Interplay Between Accessibility and Activity.” BioEssays 42, no. 4: 1900188. 10.1002/bies.201900188. 32142172

[bit70076-bib-0009] Brown, A. J. , B. Sweeney , D. O. Mainwaring , and D. C. James . 2015. “NF‐κB, CRE and YY1 Elements are Key Functional Regulators of CMV Promoter‐Driven Transient Gene Expression in Cho Cells.” Biotechnology Journal 10, no. 7: 1019–1028. 10.1002/biot.201400744. 25612069

[bit70076-bib-0010] Chan, Y. C. , E. Kienle , M. Oti , et al. 2023. “An Unbiased AAV‐STARR‐Seq Screen Revealing the Enhancer Activity Map of Genomic Regions in the Mouse Brain In Vivo.” Scientific Reports 13, no. 1: 6745. 10.1038/s41598-023-33448-w. 37185990 PMC10130037

[bit70076-bib-0011] Chatellard, P. , R. Pankiewicz , E. Meier , L. Durrer , C. Sauvage , and M. O. Imhof . 2007. “The IE2 Promoter/Enhancer Region From Mouse CMV Provides High Levels of Therapeutic Protein Expression in Mammalian Cells.” Biotechnology and Bioengineering 96, no. 1: 106–117. 10.1002/bit.21172. 16937403

[bit70076-bib-0012] Chen, J. , J. Haverty , L. Deng , et al. 2013. “Identification of a Novel Endogenous Regulatory Element in Chinese Hamster Ovary Cells by Promoter Trap.” Journal of Biotechnology 167, no. 3: 255–261. 10.1016/j.jbiotec.2013.07.001. 23850860

[bit70076-bib-0013] Dao, L. T. M. , A. O. Galindo‐Albarrán , J. A. Castro‐Mondragon , et al. 2017. “Genome‐Wide Characterization of Mammalian Promoters With Distal Enhancer Functions.” Nature Genetics 49, no. 7: 1073–1081. 10.1038/ng.3884. 28581502

[bit70076-bib-0014] Dejosez, M. , A. Dall'Agnese , M. Ramamoorthy , et al. 2023. “Regulatory Architecture of Housekeeping Genes is Driven by Promoter Assemblies.” Cell Reports 42, no. 5: 112505. 10.1016/j.celrep.2023.112505. 37182209 PMC10329844

[bit70076-bib-0015] Feichtinger, J. , I. Hernández , C. Fischer , et al. 2016. “Comprehensive Genome and Epigenome Characterization of CHO Cells in Response to Evolutionary Pressures and Over Time.” Biotechnology and Bioengineering 113, no. 10: 2241–2253. 10.1002/bit.25990. 27072894 PMC5006888

[bit70076-bib-0016] Haredy, A. M. , A. Nishizawa , K. Honda , T. Ohya , H. Ohtake , and T. Omasa . 2013. “Improved Antibody Production in Chinese Hamster Ovary Cells by ATF4 Overexpression.” Cytotechnology 65, no. 6: 993–1002. 10.1007/s10616-013-9631-x. 24026344 PMC3853642

[bit70076-bib-0017] Heintzman, N. D. , R. K. Stuart , G. Hon , et al. 2007. “Distinct and Predictive Chromatin Signatures of Transcriptional Promoters and Enhancers in the Human Genome.” Nature Genetics 39, no. 3: 311–318. 10.1038/ng1966. 17277777

[bit70076-bib-0018] Hilliard, W. , and K. H. Lee . 2021. “Systematic Identification of Safe Harbor Regions in the CHO Genome Through a Comprehensive Epigenome Analysis.” Biotechnology and Bioengineering 118, no. 2: 659–675. 10.1002/bit.27599. 33049068

[bit70076-bib-0019] Johari, Y. B. , A. J. Brown , C. S. Alves , et al. 2019. “CHO Genome Mining for Synthetic Promoter Design.” Journal of Biotechnology 294: 1–13. 10.1016/j.jbiotec.2019.01.015. 30703471

[bit70076-bib-0020] Johari, Y. B. , S. D. Estes , C. S. Alves , M. S. Sinacore , and D. C. James . 2015. “Integrated Cell and Process Engineering for Improved Transient Production of a “Difficult‐To‐Express” Fusion Protein by CHO Cells.” Biotechnology and Bioengineering 112, no. 12: 2527–2542. 10.1002/bit.25687. 26126657

[bit70076-bib-0021] Johari, Y. B. , J. M. Scarrott , T. H. Pohle , et al. 2022. “Engineering of the CMV Promoter for Controlled Expression of Recombinant Genes in HEK293 Cells.” Biotechnology Journal 17: e2200062. 10.1002/biot.202200062. 35482470

[bit70076-bib-0022] Killick, R. , and I. A. Eckley . 2014. “Changepoint: An R Package for Changepoint Analysis.” Journal of Statistical Software 58, no. 3: 1–19. 10.18637/jss.v058.i03.

[bit70076-bib-0023] Latorre, Y. , M. Torres , M. Vergara , et al. 2023. “Engineering of Chinese Hamster Ovary Cells for Co‐Overexpressing MYC and XBP1s Increased Cell Proliferation and Recombinant EPO Production.” Scientific Reports 13, no. 1: 1482. 10.1038/s41598-023-28622-z. 36707606 PMC9883479

[bit70076-bib-0024] Lee, D. , M. Shi , J. Moran , et al. 2020. “Starrpeaker: Uniform Processing and Accurate Identification of STARR‐Seq Active Regions.” Genome Biology 21, no. 1: 298. 10.1186/s13059-020-02194-x. 33292397 PMC7722316

[bit70076-bib-0025] Lee, Z. , M. Raabe , and W. S. Hu . 2021. “Epigenomic Features Revealed by ATAC‐seq Impact Transgene Expression in CHO Cells.” Biotechnology and Bioengineering 118, no. 5: 1851–1861. 10.1002/bit.27701. 33521928

[bit70076-bib-0026] Lewis, N. E. , X. Liu , Y. Li , et al. 2013. “Genomic Landscapes of Chinese Hamster Ovary Cell Lines as Revealed by the *Cricetulus griseus* Draft Genome.” Nature Biotechnology 31: 759–765. 10.1038/nbt.2624. 23873082

[bit70076-bib-0027] Lorberbaum, D. S. , and S. Barolo . 2015. “Enhancers: Holding Out for the Right Promoter.” Current Biology 25, no. 7: R290–R293. 10.1016/j.cub.2015.01.039. 25829016 PMC4824198

[bit70076-bib-0028] Majors, B. S. , N. Arden , G. A. Oyler , G. G. Chiang , N. E. Pederson , and M. J. Betenbaugh . 2008. “E2F‐1 Overexpression Increases Viable Cell Density in Batch Cultures of Chinese Hamster Ovary Cells.” Journal of Biotechnology 138, no. 3–4: 103–106. 10.1016/j.jbiotec.2008.08.003. 18778741

[bit70076-bib-0029] Muerdter, F. , Ł. M. Boryń , A. R. Woodfin , et al. 2018. “Resolving Systematic Errors in Widely Used Enhancer Activity Assays in Human Cells.” Nature Methods 15, no. 2: 141–149. 10.1038/nmeth.4534. 29256496 PMC5793997

[bit70076-bib-0030] Neumayr, C. , M. Pagani , A. Stark , and C. D. Arnold . 2019. “STARR‐Seq and UMI‐STARR‐Seq: Assessing Enhancer Activities for Genome‐Wide‐, High‐, and Low‐Complexity Candidate Libraries.” Current Protocols in Molecular Biology 128, no. 1: e105. 10.1002/cpmb.105. 31503413 PMC9286403

[bit70076-bib-0031] Nguyen, L. N. , M. Baumann , H. Dhiman , et al. 2019. “Novel Promoters Derived From Chinese Hamster Ovary Cells via In Silico and In Vitro Analysis.” Biotechnology Journal 14, no. 11: e1900125. 10.1002/biot.201900125. 31271264

[bit70076-bib-0032] Nishimiya, D. , T. Mano , K. Miyadai , H. Yoshida , and T. Takahashi . 2013. “Overexpression of CHOP Alone and in Combination With Chaperones is Effective In Improving Antibody Production in Mammalian Cells.” Applied Microbiology and Biotechnology 97, no. 6: 2531–2539. 10.1007/s00253-012-4365-9. 22926643

[bit70076-bib-0033] O'Callaghan, P. M. , and Y. B. Johari . 2025. “Optimizing Expression Vectors for Biotherapeutic Production: Design Principles, Titer‐Influencing Factors, and Promoters.” BioProcess International 23, no. 4: 12–19.

[bit70076-bib-0034] Panigrahi, A. , and B. W. O'Malley . 2021. “Mechanisms of Enhancer Action: The Known and the Unknown.” In Genome Biology 22, 108, Issue 1. BioMed Central Ltd. 10.1186/s13059-021-02322-1. 33858480 PMC8051032

[bit70076-bib-0035] Policarpi, C. , M. Munafò , S. Tsagkris , V. Carlini , and J. A. Hackett . 2024. “Systematic Epigenome Editing Captures the Context‐Dependent Instructive Function of Chromatin Modifications.” Nature Genetics 56, no. 6: 1168–1180. 10.1038/s41588-024-01706-w. 38724747 PMC11176084

[bit70076-bib-0036] Rotondaro, L. , A. Mele , and G. Rovera . 1996. “Efficiency of Different Viral Promoters in Directing Gene Expression in Mammalian Cells: Effect of 3′‐Untranslated Sequences.” Gene 168, no. 2: 195–198. 10.1016/0378-1119(95)00767-9. 8654943

[bit70076-bib-0037] Smith, G. D. , W. H. Ching , P. Cornejo‐Páramo , and E. S. Wong . 2023. “Decoding Enhancer Complexity With Machine Learning and High‐throughput Discovery.” In Genome Biology (24, Issue 1). BioMed Central Ltd. 10.1186/s13059-023-02955-4. PMC10176946 37173718

[bit70076-bib-0038] Tastanova, A. , A. Schulz , M. Folcher , et al. 2016. “Overexpression of YY1 Increases the Protein Production in Mammalian Cells.” Journal of Biotechnology 219: 72–85. 10.1016/j.jbiotec.2015.12.005. 26686315

[bit70076-bib-0039] Thul, P. J. , and C. Lindskog . 2018. “The Human Protein Atlas: A Spatial Map of the Human Proteome.” Protein Science 27, no. 1: 233–244. 10.1002/pro.3307. 28940711 PMC5734309

[bit70076-bib-0040] Thurman, R. E. , E. Rynes , R. Humbert , et al. 2012. “The Accessible Chromatin Landscape of the Human Genome.” Nature 489: 75–82. 10.1038/nature11232. 22955617 PMC3721348

[bit70076-bib-0041] Vanhille, L. , A. Griffon , M. A. Maqbool , et al. 2015. “High‐Throughput and Quantitative Assessment of Enhancer Activity in Mammals by CapSTARR‐Seq.” Nature Communications 6: 6905. 10.1038/ncomms7905. 25872643

[bit70076-bib-0042] Walsh, G. , and E. Walsh . 2022. “Biopharmaceutical Benchmarks 2022.” Nature Biotechnology 40: 1722–1760. 10.1038/s41587-022-01582-x. PMC9735008 36471135

[bit70076-bib-0043] Weintraub, A. S. , C. H. Li , A. V. Zamudio , et al. 2017. “YY1 is a Structural Regulator of Enhancer‐Promoter Loops.” Cell 171: 1573–1588.e28. 10.1016/j.cell.2017.11.008. 29224777 PMC5785279

[bit70076-bib-0044] Wu, F. , and A. S. Lee . 2001. “YY1 as a Regulator of Replication‐Dependent Hamster Histone H3.2 Promoter and an Interactive Partner of AP‐2.” Journal of Biological Chemistry 276, no. 1: 28–34. 10.1074/jbc.M006074200. 11018030

[bit70076-bib-0045] Wurm, M. J. , and F. M. Wurm . 2021. “Naming Cho Cells for Bio‐Manufacturing: Genome Plasticity and Variant Phenotypes of Cell Populations in Bioreactors Question the Relevance of Old Names.” Biotechnology Journal 16, no. 7: e2100165. 10.1002/biot.202100165. 34050613

[bit70076-bib-0046] Zabidi, M. A. , C. D. Arnold , K. Schernhuber , et al. 2015. “Enhancer‐Core‐Promoter Specificity Separates Developmental and Housekeeping Gene Regulation.” Nature 518, no. 7540: 556–559. 10.1038/nature13994. 25517091 PMC6795551

[bit70076-bib-0047] Zhu, I. , W. Song , I. Ovcharenko , and D. Landsman . 2021. “A Model of Active Transcription Hubs That Unifies the Roles of Active Promoters and Enhancers.” Nucleic Acids Research 49, no. 8: 4493–4505. 10.1093/nar/gkab235. 33872375 PMC8096258

