# Supplementary material for: Comprehensive Mapping of Functional Enhancers in Chinese Hamster Ovary Cells
Source: Biotechnol Bioeng. 2025 Oct 3;123(1):196–211. doi: 10.1002/bit.70076 (PMC12699134; doi:10.1002/bit.70076)
Supplement: Supplementary file 1 — Figure S1: Schematic representation of maxGFP reporter vectors. Figure S2: Schematic representation of mCMV and Ori STARR‐seq vectors. Figure S3: Comparison in STARR‐seq activity between samples collected at 6 hr and 24 hr post‐transfection. Figure S4: Comparison in STARR‐seq activity quantification between mCMV and Ori as core promoters. Figure S5: DeepSTARR model performance and nucleotide contribution prediction. [file BIT-123-196-s001.pdf]

SUPPLEMENTARY FIGURES

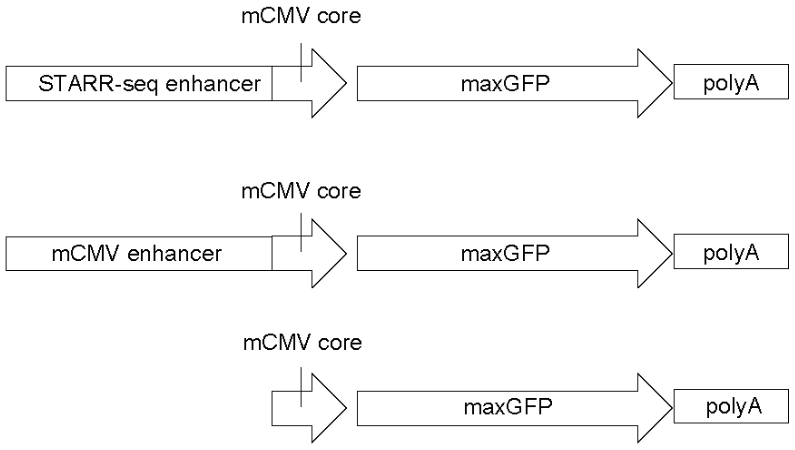

Figure S1. Schematic representation of maxGFP reporter vectors.

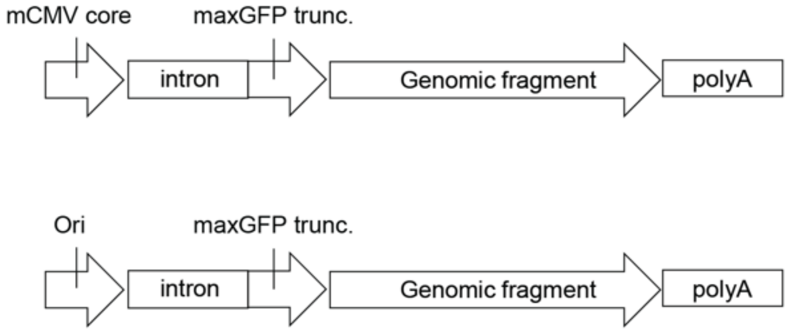

Figure S2. Schematic representation of mCMV and Ori STARR-seq vectors.

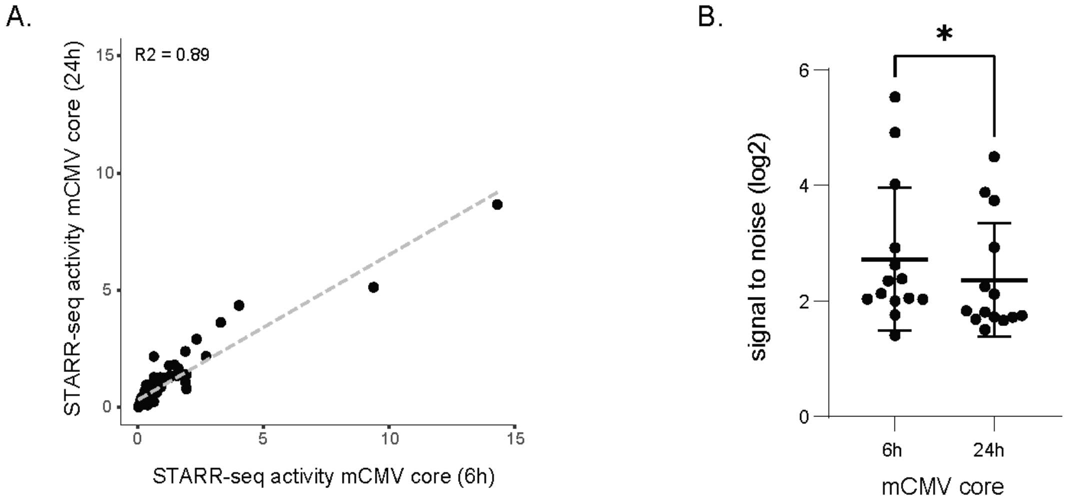

Figure S3. Comparison in STARR-seq activity between samples collected at 6 hr and 24 hr post-transfection. Signal-to-noise equals the average activity of high confidence positive enhancer sequence (*n* = 14) over average activity of negative control sequence (*n* = 7) (Muerdter et al., 2018). *p-value < 0.05 (two-sided paired t-test).

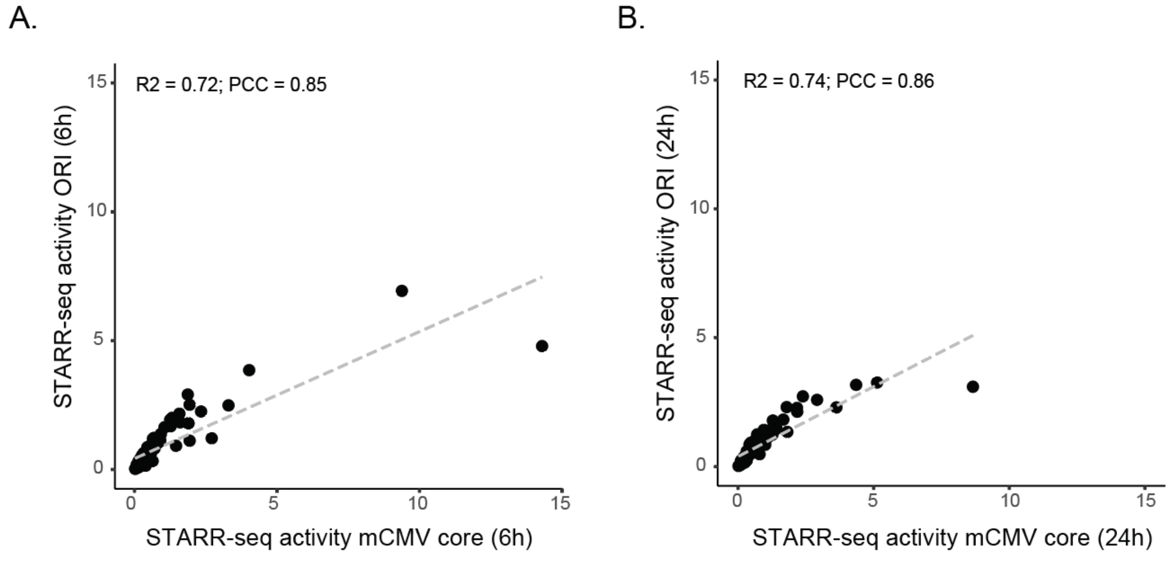

Figure S4. Comparison in STARR-seq activity quantification between mCMV and Ori as core promoters. Correlation of STARR-seq activity measured with mCMV core and Ori-based screening vectors at 6 hr **(A)** and 48 hr **(B)** post-transfection for 2 biological replicates. Shown are fragment counts for 500 bp genomic windows, normalised to reads per million.

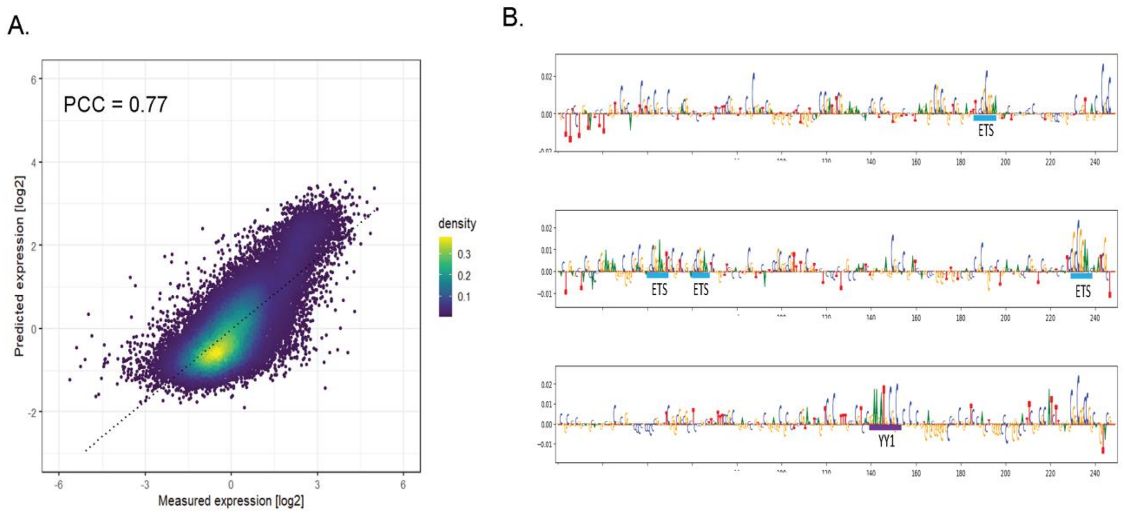

Figure S5. DeepSTARR model performance and nucleotide contribution prediction. **A.** Scatter plot showing the relationship between predicted and observed enhancer activity signals for all DNA sequences in the test dataset. **B.** DeepSTARR nucleotide contribution scores for three representative sequences with high predicted STARR-seq activity. Instances of ETS and YY1 binding motifs are highlighted. PCC: Pearson correlation coefficient.
